# Moonlighting proteins are variably exposed at the cell surfaces of *Candida glabrata*, *Candida parapsilosis* and *Candida tropicalis* under certain growth conditions

**DOI:** 10.1186/s12866-019-1524-5

**Published:** 2019-07-03

**Authors:** Justyna Karkowska-Kuleta, Dorota Satala, Oliwia Bochenska, Maria Rapala-Kozik, Andrzej Kozik

**Affiliations:** 1 Department of Comparative Biochemistry and Bioanalytics, Faculty of Biochemistry, Biophysics and Biotechnology, Jagiellonian University, Gronostajowa 7, 30-387, Krakow, Poland; 2 Department of Analytical Biochemistry, Faculty of Biochemistry, Biophysics and Biotechnology, Jagiellonian University, Gronostajowa 7, 30-387, Kraków, Poland

**Keywords:** Non-albicans *Candida* species, Candidiasis, Cell wall, Enolase, Cell surface proteome

## Abstract

**Background:**

Adaptability to different environmental conditions is an essential characteristic of pathogenic microorganisms as it facilitates their invasion of host organisms. The most external component of pathogenic yeast-like fungi from the *Candida* genus is the multilayered cell wall. This structure is composed mainly of complex polysaccharides and proteins that can undergo dynamic changes to adapt to the environmental conditions of colonized niches.

**Results:**

We utilized cell surface shaving with trypsin and a shotgun proteomic approach to reveal the surface-exposed proteins of three important non-albicans *Candida* species—*C. glabrata*, *C. parapsilosis* and *C. tropicalis*. These proteinaceous components were identified after the growth of the fungal cells in various culture media, including artificial saliva, artificial urine and vagina-simulative medium under aerobic conditions and anaerobically in rich YPD medium. Several known proteins involved in cell wall maintenance and fungal pathogenesis were identified at the cell surface as were a number of atypical cell wall components—pyruvate decarboxylase (Pdc11), enolase (Eno1) and glyceraldehyde-3-phosphate dehydrogenase (Tdh3) which are so-called ‘moonlighting’ proteins. Notably, many of these proteins showed significant upregulation at the cell surface in growth media mimicking the conditions of infection compared to defined synthetic medium.

**Conclusions:**

Moonlighting proteins are expressed under diverse conditions at the cell walls of the *C. glabrata*, *C. parapsilosis* and *C. tropicalis* fungal pathogens. This indicates a possible universal surface-associated role of these factors in the physiology of these fungi and in the pathology of the infections they cause*.*

**Electronic supplementary material:**

The online version of this article (10.1186/s12866-019-1524-5) contains supplementary material, which is available to authorized users.

## Background

Pathogenic microorganisms possess the important ability to adapt readily to different environmental conditions, which they exploit when colonizing new infectious niches in a host organism. During the initiation and development of the infectious process, these microbes typically have to contend with changes in temperature, pH, and osmolarity, the variable availability of oxygen and nutrients, oxidative stress and the host immune response. This may require not only the use of an entire suite of classic virulence factors, such as hydrolytic enzymes, toxins or adhesins, but also other less obvious or not well-known mechanisms that enable microbial survival in the face of adverse environmental conditions. One such unusual mechanism may be cell surface enzymes that originate in the cytoplasm in the pathogens and are primarily involved in essential intracellular metabolic processes such as glycolysis, the citric acid cycle or the pentose phosphate pathway [1]. A cellular location that is completely different from the original suggests an alternate function for such factors that is not caused by gene fusions or splicing variations. The term “moonlighting proteins” was coined as a result [2]. The functional strategy involved in this instance is successfully used by many species of pathogenic bacteria in which surface-exposed moonlighting proteins play an important role in the process of adhesion to host cells and tissues, binding of numerous proteinaceous targets within the host organism, and evasion of the immune system [3, 4].

This phenomenon has also been described for yeast-like fungi from the *Candida* genus. These microbes have the potential to be dangerous pathogens in humans that can cause not only frequently occurring superficial infections of the skin and mucosal surfaces, but also invasive, systemic infections, especially in a host with impaired immunity [5, 6]. Several moonlighting proteins exposed on the cell surface of *C. albicans*, *C. tropicalis* and *C. parapsilosis* have been identified as binding partners for human extracellular matrix proteins [7, 8], plasminogen and components of the complement system and kinin-generating system [9–14], and even as molecules that mediate the binding of fungal cells to human cells [15]. Moreover, moonlighting proteins from different *Candida* species can play a role in the adhesion of fungi to medical devices made of silicone or polyvinyl chloride [16].

A potential protective role of surface-exposed moonlighting proteins has recently been proposed as a response to oxidative stress caused by H_2_O_2_ in *C. albicans*, *C. glabrata*, *C. krusei* and *C. parapsilosis*. It was demonstrated that several metabolic enzymes present at the cell wall of these fungi grown in liquid media or in sessile cells forming biofilms might represent a primary line of fungal defense against reactive oxygen species generated during phagocytic respiratory burst in conjunction with typical antioxidant systems [17, 18]. This particular mechanism, in tandem with the ability to adhere to different biotic and abiotic surfaces and to influence the action of important plasma proteolytic cascades, can significantly contribute to the virulence of *Candida* fungi. The invasion of the host organism and colonization of new niches by fungal cells undoubtedly require a dynamic adaptation of these microorganisms to new environmental conditions, including changes to their cell wall proteome [19]. However, given that significant changes in the frequency of infections caused by particular *Candida* species have been observed and that species other than *C. albicans*, mainly *C. glabrata*, *C. parapsilosis* and *C. tropicalis*, have been indicated as the cause of superficial and invasive candidiasis [20–22], particular attention should be paid to the pathogenicity-related attributes of these other species, as they are increasingly reported to be a major threat for individuals with impaired immunity. For *C. albicans*, the cell wall structure and changes in its proteome is relatively well characterized but there have been less reports on the composition of the cell walls of other species of *Candida* genus. Among the proteins present at the surface of *C. albicans* cells, many moonlighting proteins have been repeatedly described, including enolase (Eno1), glyceraldehyde-3-phosphate dehydrogenase (Tdh3), alcohol dehydrogenase (Adh1), phosphoglycerate kinase (Pgk1), transaldolase (Tal1), pyruvate decarboxylase (Pdc11) and others [23–25]. As significant differences in virulence attributes between species can be expected, including a variability of protein exposition at the cell surface, this may result in discrepancies in pathogen-host interactions, the increase of incidence of candidiases caused by species other than the well described *C. albicans* and greater difficulties in the treatment of such infections. Hence, the aim of our current study was to investigate overall changes in the surface exposure of cell wall-associated proteins in *C. glabrata*, *C. parapsilosis* and *C. tropicalis*. We did these analyses under growth conditions mimicking the microenvironment at different body sites that could potentially be colonized by these pathogens, with a particular emphasis on the contribution of moonlighting proteins to the entire repertoire exposed on the candidal cell surface.

## Results

### Diverse proteins present on the cell surfaces of *C. glabrata*, *C. parapsilosis* and *C. tropicalis* depending on the growth conditions

The detection and quantification of the proteins expressed on the *C. glabrata*, *C. parapsilosis* or *C. tropicalis* cell surface involved the tryptic digestion of these factors without disturbing the integrity of the cells followed by peptide separation and identification via LC-MS/MS (high performance liquid chromatography-coupled tandem mass spectrometry) [26].

We analyzed three biological replicates and only further evaluated the proteins that were detected at least twice. In total, 53 proteins for *C. glabrata*, 37 for *C. parapsilosis* and 13 for *C. tropicalis* were identified at the fungal cell surface under all of the tested growth conditions. Notably however, a significant degree of quantitative and qualitative diversity was evident depending on the culture media used (Additional file 1: Table S1, Additional file 2: Table S2, Additional file 3: Table S3 and Additional file 4: Table S4). The highest number of proteins was detected for each species when using YPD medium under anaerobic conditions (AN) (29, 27 and 11 for *C. glabrata*, *C. parapsilosis* and *C. tropicalis*, respectively). The lowest number of *C. glabrata* proteins (8) and *C. parapsilosis* proteins (6) was detected after growth in vagina-simulative medium (VS), and for *C. tropicalis* following cultivation in artificial urine (AU) (1).

**Fig. 1:**
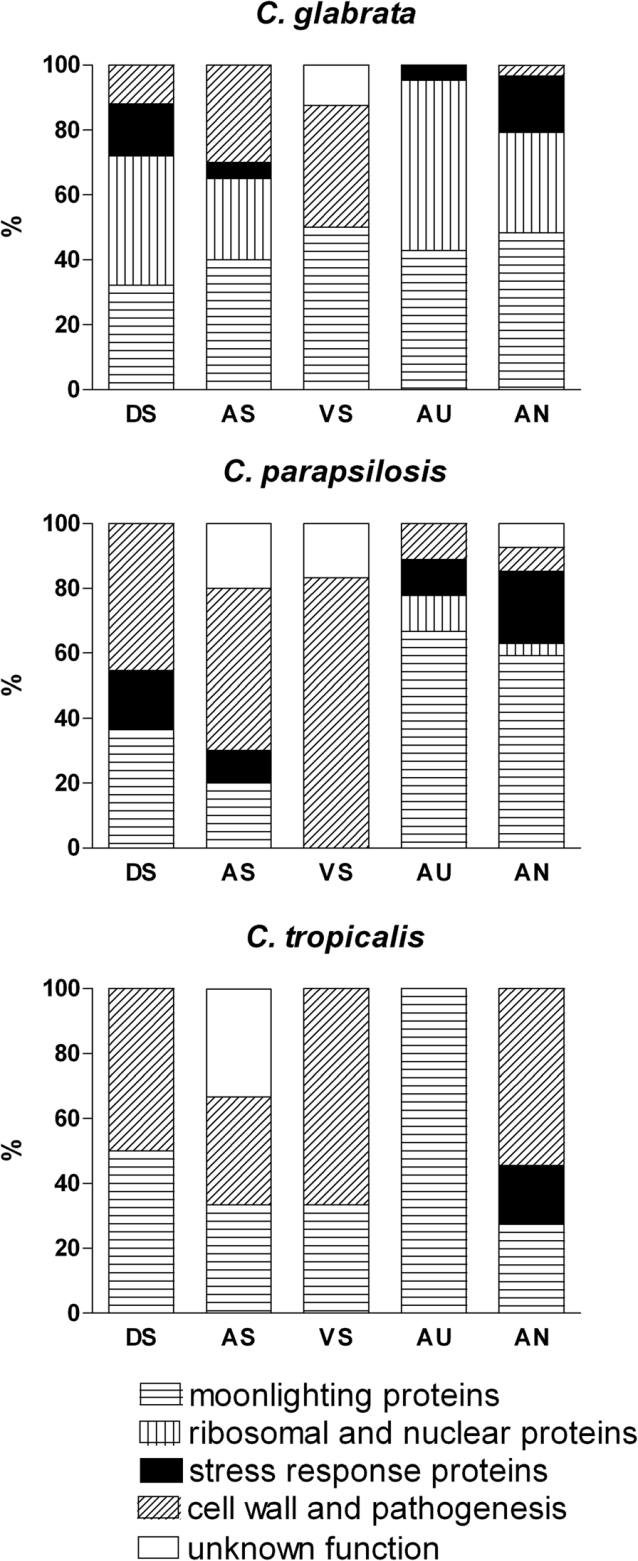
Distribution of surface-exposed fungal proteins and their division into major functional groups**.** *C. glabrata, C. parapsilosis* and *C. tropicalis* cells were cultured aerobically in different growth media at 37 °C for 16 h (DS, an amino acid-based, chemically defined liquid synthetic medium; AS, artificial saliva, VS, vagina-simulative medium, and AU, artificial urine) or cultured for 72 h in YPD medium under anaerobic conditions (AN). Surface-exposed proteins were identified by cell surface shaving with trypsin followed by liquid chromatography coupled to tandem mass spectrometry. The protein classifications were made on the basis of the Gene Ontology (GO) annotations (molecular function and involvement in similar cellular processes) from the *Candida* Genome Database (CGD) and *Saccharomyces* Genome Database (SGD)

The use of different culture conditions, principally the composition of the medium, a pH range from 4.2 to 7.0, and a variation in oxygen availability (normoxia vs. anoxia), allowed the identification of several groups of proteins exposed at the surface of *C. glabrata*, *C. parapsilosis* and *C. tropicalis* cells. These functionally diverse groups were distinguished by their gene ontology (GO) annotations with regard to the particular molecular and biological processes in which they were involved. Appropriate GO annotations for each of *C. glabrata*, *C. parapsilosis* or *C. tropicalis* orthologous genes were assigned based on the information from the *Candida* Genome Database (CGD, http://www.candidagenome.org) [27] or *Saccharomyces* Genome Database (SGD, https://www.yeastgenome.org) [28]. Five major groups of proteins were thereby specified, including (i) typical cell wall proteins and secreted proteins equipped with a signal peptide, and involved in cell wall maintenance and fungal pathogenesis, (ii) stress response proteins, (iii) atypical cell wall proteins, i.e. moonlighting proteins, (iv) ribosomal and nuclear proteins, and (v) proteins of unknown function. Under the culture conditions used in our experiments, we observed a large variation between individual groups of proteins detected after growth in specific media, and found that not all groups were represented when different media were used (Fig. 1).

**Fig. 2:**
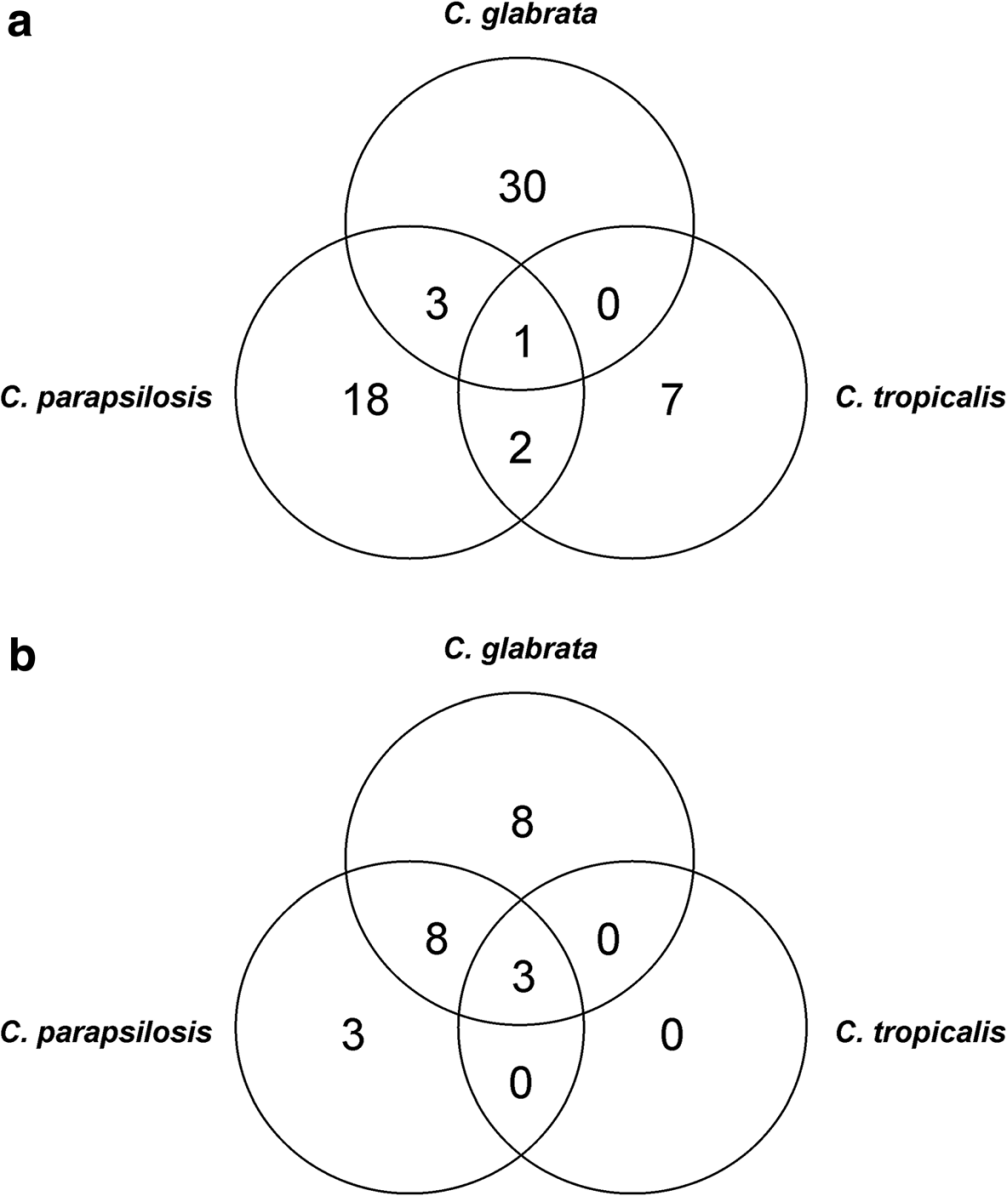
Venn diagrams indicating the number of surface-exposed orthologous proteins shared between *Candida* species***.*** *C. glabrata, C. parapsilosis* and *C. tropicalis* cells were cultured for 16 or 72 h at 37 °C and then cell surface shaved with trypsin to identify surface-exposed proteins via LC-MS/MS. The functions of the identified proteins were assigned on the basis of GO annotations from the CGD and SGD. The numbers of shared or exclusive orthologous proteins identified in *C. glabrata, C. parapsilosis* and *C. tropicalis* were compared according to the two major groups of proteins identified under all tested growth conditions i.e. **a**, typical cell wall proteins, stress response proteins, ribosomal and nuclear proteins and proteins with unknown function; and **b**, moonlighting proteins, that are metabolic enzymes primarily involved in essential intracellular metabolic processes

The lowest level of functional variability in *C. glabrata* and *C. parapsilosis* was observed after growth in vagina-simulative medium. Only typical cell wall proteins and secreted proteins were identified for these species under these conditions with some additional moonlighting proteins for *C. glabrata,* together with a few proteins of unknown function in both species. For *C. tropicalis*, the functional diversity of the identified proteins was generally reduced compared to the other two non-albicans *Candida* species. This was consistent with our observation of a generally smaller number of surface proteins detected for *C. tropicalis* under our current experimental culture conditions. Indeed, only one surface-exposed moonlighting protein was detectable in *C. tropicalis* using our method after growth in artificial urine. Moonlighting proteins were detected at the surfaces of almost all of our tested species grown in almost every culture medium used in our present analysis, and then accounted for at least 25% of a total protein pool. The one exception was *C. parapsilosis* cultivated in vagina-simulative medium. A more detailed qualitative comparison of the similarities and differences between the complete sets of cell surface proteins in the three investigated *Candida* species was conducted to determine the number of orthologous proteins shared between them (Fig. 2). When only moonlighting proteins and all other surface-exposed proteins were analyzed, three atypical cell wall proteins (Pdc11, Eno1 and Tdh3) were found to be shared by all three tested species and eight by *C. parapsilosis* and *C. glabrata*. Moreover, three and eight moonlighting proteins were identified to be exclusive to the first and latter species, respectively. Analogous analysis of the second separate group containing all other cell wall proteins showed in contrast to the first group that only one orthologous protein was shared by all three investigated species (the cell wall mannoprotein Mp65). Three orthologous proteins were shared by *C. glabrata* and *C. parapsilosis* (heat shock protein Ssa2, heat shock protein Hsp12, elongation factor 2) and two by the latter species and *C. tropicalis* (yeast-form wall protein Ywp1 and inducible acid phosphatase Pho100). In addition, more proteins from this second group were indicated to be unique to individual species, i.e., 7 for *C. tropicalis*, 18 for *C. parapsilosis* and 30 for *C. glabrata*.

### Expression changes in moonlighting proteins at the cell surfaces of *C. glabrata*, *C. parapsilosis* and *C. tropicalis*

Considering a potentially important function of moonlighting proteins in fungal pathogenesis and adhesion, an unusual route of their secretion and interspecies differences and similarities in their distribution, further attention was focused only on this particular group of surface-exposed proteins. On the basis of GO annotations concerning the molecular functions and biological processes assigned to cell wall proteins, expression changes in moonlighting proteins at the surface of *C. glabrata*, *C. parapsilosis* or *C. tropicalis* cells were quantitatively analyzed. Four types of culture media of a different composition and pH were employed as they simulate different potential sites of infection such as the oral cavity (artificial saliva, AS, pH 7.0) [29], vaginal microenvironment (vagina-simulative medium, VS, pH 4.2) [30], the urinary tract (artificial urine, AU, pH 5.8) [31] and gastrointestinal tract (YPD medium under anaerobic conditions) [32–34]. A synthetic medium (DS) with a particular amino acid composition [35] was used as a reference to compare the protein groups present at the fungal cell wall under the different culture conditions. In the analysis, normalized spectral abundance factors (NSAFs), that quantitatively indicate the level of each moonlighting protein at the cell surface in different media, were calculated and analyzed in terms of statistically significant differences (Tables 1, 2 and 3). In *C. glabrata*, eight moonlighting proteins were detected after growth both in DS and in at least one other medium, and a significant increase in the amount of five proteins at the cell surface was also observed in relation to DS conditions. Moreover, 11 additional moonlighting proteins were detected in *C. glabrata* alone after growth in AS, VS, AU or anaerobic conditions, but not in the synthetic medium. In *C. parapsilosis*, only four moonlighting proteins were detected in DS cultures, and an additional 10 proteins were identified in this microbe after growth in AS, AU or AN. No moonlighting proteins were found at the *C. parapsilosis* cell surface after growth in VS medium. Moreover, only three moonlighting factors were detected at the surface of *C. tropicalis* cells (Pdc11, Eno1 and Tdh3) when this fungus was grown in DS medium and in AN. Notably also, an increased abundance of individual moonlighting proteins at the cell surface was observed mainly after growth in other media under aerobic conditions (Eno1 in AS and Tdh3 in VS), but not under anaerobic conditions. Relative differences in the level of expression of selected surface-exposed moonlighting proteins are shown in Additional file 5: Figure S1.

**Table 1:** Mass spectrometry identification of *C. glabrata* moonlighting proteins present at the cell surface under different growth conditions

NCBI accession number	Protein description	defined synthetic medium (DS)	artificial saliva (AS)	vagina-simulative medium (VS)	artificial urine (AU)	anaerobic conditions (AN)
gi|50,288,681 (XP_446770)	uncharacterized protein CAGL0G09383g [*Candida glabrata*] glyceraldehyde-3-phosphate dehydrogenase 3 (Tdh3)	0.10203	0.06654 ^ns^	0.26374*	0.10739 ^ns^	0.08619 ^ns^
gi|50,289,857 (XP_447360)	uncharacterized protein CAGL0I02486g [*Candida glabrata*] enolase I (Eno1)	0.04892	0.08869*	0.18915 ^ns^	0.08158*	0.12634*
gi|50,293,403 (XP_449113)	uncharacterized protein CAGL0L07722g [*Candida glabrata*] phosphoglycerate kinase (Pgk1)	0.03511	0.03084 ^ns^	–	0.03684 ^ns^	0.05591 ^ns^
gi|50,287,073 (XP_445966)	uncharacterized protein CAGL0E06358g [*Candida glabrata*] phosphoglycerate mutase 1 (Gpm1)	0.02871	–	–	–	0.02756 ^ns^
gi|50,285,355 (XP_445106)	uncharacterized protein CAGL0B03069g [*Candida glabrata*] transaldolase (Tal1)	0.01863	0.03913*	–	–	0.01621 ^ns^
gi|50,294,908 (XP_449865)	uncharacterized protein CAGL0M12034g [*Candida glabrata*] pyruvate kinase (Cdc19)	0.01426	–	–	0.05353****	0.03152*
gi|50,292,893 (XP_448879)	uncharacterized protein CAGL0L02497g [*Candida glabrata*] fructose-bisphosphate aldolase (Fba1)	0.01149	–	–	–	0.04445*
gi|50,295,024 (XP_449923)	uncharacterized protein CAGL0M13343g [*Candida glabrata*] 6-phosphogluconate dehydrogenase (Gnd1)	0.00730	–	–	0.01415 ^ns^	
gi|25,992,752 (AAN77243)	pyruvate decarboxylase (Pdc11) [*Candida glabrata*]	–	–	0.05196^↑^	0.08659^↑^	0.02002^↑^
gi|50,286,085 (XP_445471)	uncharacterized protein CAGL0D01298g [*Candida glabrata*] transketolase (Tkl1)	–	0.01866^↑^	–	–	0.01398^↑^
gi|50,289,205 (XP_447033)	uncharacterized protein CAGL0H05445g [*Candida glabrata*] glucose-6-phosphate isomerase (Pgi1)	–	0.02312^↑^	–	–	–
gi|50,289,307 (XP_447084)	uncharacterized protein CAGL0H06633g [*Candida glabrata*] phosphoenolpyruvate carboxykinase (Pck1)	–	–	–	0.03884^↑^	–
gi|50,289,459 (XP_447161)	uncharacterized protein CAGL0H08327g [*Candida glabrata*] triosephosphate isomerase (Tpi1)	–	0.07053^↑^	–	–	0.03771^↑^
gi|50,289,591 (XP_447227)	uncharacterized protein CAGL0H09878g [*Candida glabrata*] inorganic pirophosphatase (Ipp1)	–	–	–	–	0.03773^↑^
gi|50,290,317 (XP_447590)	uncharacterized protein CAGL0I07843g [*Candida glabrata*] alcohol dehydrogenase I (Adh1)	–	–	–	0.04322^↑^	0.03855^↑^
gi|50,291,871 (XP_448368)	uncharacterized protein CAGL0K03289g [*Candida glabrata*] glucose-6-phosphate 1-epimerase (Gpe1)	–	–	–	–	0.00787^↑^
gi|50,292,233 (XP_448549)	uncharacterized protein CAGL0K07546g [*Candida glabrata*] probable phosphoglycerate mutase (Pmu1)	–	–	0.31504^↑^	–	–
gi|50,293,881 (XP_449352)	uncharacterized protein CAGL0M00176g [*Candida glabrata*] branched-chain-amino-acid aminotransferase (Twt2)	–	0.01708^↑^	–	–	–
gi|50,294,878 (XP_449850)	uncharacterized protein CAGL0M11704g [*Candida glabrata*] alkyl hydroperoxide reductase (Ahp1)	–	–	–	–	0.03156^↑^

Cell surface shaving of *C. glabrata* cultures with trypsin and the additional digestion of the obtained proteins for 24 h was performed. The resulting peptides were analyzed using the Dionex Ultimate 3000 UHPLC system coupled to an HCTUltra ETDII mass spectrometer. The obtained lists of peaks were searched against the NCBI protein database using an in-house Mascot server. The normalized abundance factors (NSAFs) were calculated for each of the tested growth conditions and the statistical significance with respect to the defined synthetic medium is indicated as follows: *p values from 0.01 to 0.05; ****p < 0.0001; ns, not significant by Student *t*-test. The arrows indicate that the protein was only identified from cultures grown in AS, VS, AU or AN and not in the synthetic medium

**Table 2:** Mass spectrometry identification of *C. parapsilosis* moonlighting proteins present at the cell surface under different growth conditions

NCBI accession number	Protein description	defined synthetic medium (DS)	artificial saliva (AS)	vagina-simulative medium (VS)	artificial urine (AU)	anaerobic conditions (AN)
gi|354,546,348 (CCE43078)	hypothetical protein CPAR2_207210 [*Candida parapsilosis*] enolase (Eno1)	0.07214	–	–	0.09507 ^ns^	0.06579 ^ns^
gi|354,545,590 (CCE42318)	hypothetical protein CPAR2_808670 [*Candida parapsilosis*] glyceraldehyde-3-phosphate dehydrogenase (Tdh3)	0.07167	–	–	0.11546 ^ns^	0.07485 ^ns^
gi|354,545,888 (CCE42617)	hypothetical protein CPAR2_202600 [*Candida parapsilosis*] transaldolase (Tal1)	0.06634	–	–	0.08495 ^ns^	0.02001**
gi|354,547,143 (CCE43876)	hypothetical protein CPAR2_501020 [*Candida parapsilosis*] pyruvate decarboxylase (Pdc11)	0.01985	–	–	0.03279***	0.02973 ^ns^
gi|354,543,158 (CCE39876)	hypothetical protein CPAR2_602950 [*Candida parapsilosis*] phosphoglycerate kinase (Pgk1)	–	–	–	0.12601^↑^	0.08621^↑^
gi|354,543,177 (CCE39895)	hypothetical protein CPAR2_603140 [*Candida parapsilosis*] putative ketol-acid reductoisomerase (Ilv5)	–	–	–	–	0.01782^↑^
gi|354,543,405 (CCE40124)	hypothetical protein CPAR2_101620 [*Candida parapsilosis*] acetyl-coA hydrolase (Ach1)	–	–	–	–	0.01021^↑^
gi|354,545,521 (CCE42249)	hypothetical protein CPAR2_807980 [*Candida parapsilosis*] triosephosphate isomerase (Tpi)	–	–	–	–	0.04040^↑^
gi|354,545,980 (CCE42709)	hypothetical protein CPAR2_203520 [*Candida parapsilosis*] 6-phosphogluconate dehydrogenase (Gnd1)	–	–	–	–	0.01057^↑^
gi|354,546,116 (CCE42845)	hypothetical protein CPAR2_204880 [*Candida parapsilosis*] phosphoglucose isomerase (Pgi1)	–	–	–	–	0.01127^↑^
gi|354,546,805 (CCE43537)	hypothetical protein CPAR2_211810 [*Candida parapsilosis*] phosphoglycerate mutase (Gpm1)	–	–	–	–	0.07052^↑^
gi|354,546,845 (CCE43577)	hypothetical protein CPAR2_212210 [*Candida parapsilosis*] NAD-aldehyde dehydrogenase (Ald5)	–	0.23428^↑^	–	–	
gi|354,547,299 (CCE44033)	hypothetical protein CPAR2_502580 [*Candida parapsilosis*] alcohol dehydrogenase (Adh1)	–	0.13296^↑^	–	0.08365^↑^	0.13991^↑^
gi|354,547,586 (CCE44321)	hypothetical protein CPAR2_401230 [*Candida parapsilosis*] fructose-bisphosphate aldolase (Fba1)	–	–	–	–	0.04268^↑^

Cell surface shaving of *C. parapsilosis* cultures with trypsin and additional digestion of the obtained proteins for 24 h was performed. The resulting peptides were then analyzed using the Dionex Ultimate 3000 UHPLC system coupled to an HCTUltra ETDII mass spectrometer. The obtained lists of peaks were searched against the NCBI protein database using an in-house Mascot server. The normalized abundance factors (NSAFs) were calculated for each of the tested growth conditions and the statistical significance with respect to the defined synthetic medium is indicated as follows: **p from 0.001 to 0.01, ***p from 0.0001 to 0.001; and ns, not significant by Student *t*-test. The arrows indicate that the protein was only identified from cultures grown in AS, VS, AU or AN and not in the synthetic medium

**Table 3:** Mass spectrometry identification of *C. tropicalis* moonlighting proteins present at the cell surface after growth under different conditions

NCBI accession number	Protein description	defined synthetic medium (DS)	artificial saliva (AS)	vagina- simulative medium (VS)	artificial urine (AU)	anaerobic conditions (AN)
gi|255,727,881 (XP_002548866)	enolase 1 (Eno1) CTRG_03163 [*Candida tropicalis* MYA-3404]	0.08812	0.27053**	–	0.32363**	0.15934 ^ns^
gi|255,729,208 (XP_002549529)	pyruvate decarboxylase (Pdc11) CTRG_03826 [*Candida tropicalis* MYA-3404]	0.04341	–	–	–	0.03722 ^ns^
gi|255,732,890 (XP_002551368)	glyceraldehyde-3-phosphate dehydrogenase (Tdh3) CTRG_05666 [*Candida tropicalis*MYA-3404]	0.09736	–	0.57733****	–	0.09534 ^ns^

Cell surface shaving of *C. tropicalis* cultures with trypsin and additional digestion of the obtained proteins for 24 h was performed. The resulting peptides were then analyzed using the Dionex Ultimate 3000 UHPLC system coupled to an HCTUltra ETDII mass spectrometer. The obtained lists of peaks were searched against the NCBI protein database using an in-house Mascot server. The normalized abundance factors (NSAFs) were calculated for each of the tested growth conditions and the statistical significance with respect to the defined synthetic medium is indicated as follows:**p from 0.001 to 0.01; ****p < 0.0001; and ns, not significant by Student *t*-test

## Discussion

The cell wall of fungi from the *Candida* genus is not a rigid, static structure, but it is an important external organelle of the cell that is subjected to dynamic changes in response to various environmental conditions. Composed mainly of N- and O-mannosylated proteins, other proteins, β-1,3- and β-1,6-glucans, chitin and small amounts of lipids, the cell wall is a part of the pathogenic fungal microbe that has an immediate and constant contact with the host during infection. Hence, it can play not only a protective role against host immune defenses and environmental stress stimuli, but also an active part in the pathogenesis of infections via cell surface virulence factors [36]. Although the general cell wall structure is similar among the species within the genus *Candida*, there are differences in this composition between different species and strains, or even different morphological forms of fungi [37, 38]. In our previous study, we reported differences between the sets of proteins exposed at the surface of *C. parapsilosis* and *C. tropicalis* cells grown in YPD or RPMI 1640 culture media that affected the fungal morphology and were indicative of a large diversity among these factors [26].

There are several distinct groups among the known candidal cell wall proteins. Covalently bound fungal cell wall proteins are highly glycosylated, equipped with a motif that facilitates a classical secretion process and are linked to cell wall polysaccharides or other cell wall proteins via a glycosylphosphatidylinositol (GPI) anchor, alkali-sensitive linkages or disulfide bridges. However, a number of atypical proteins of cytoplasmic origin are also present at the surface of *Candida* cells [39, 40]. These proteins of an undefined way of secretion, that play a completely different role at the cell surface than in the cytoplasm are known as moonlighting proteins and are increasingly considered to be important factors in fungal virulence [41]. One hypothesis is that these evolutionary conserved proteins become exposed at the cell wall of a fungal pathogen and function in molecular mimicry pathways. This possibility is due to the high similarity of these factors with host proteins which will help the invading microbes to thwart the host’s immune system [42].

With the use of a cell surface shaving method with trypsin in combination with a shotgun proteomic approach, we have here identified cell surface proteins from three species from the *Candida* genus—*C. glabrata*, *C. parapsilosis* and *C. tropicalis*. These fungi were grown in four different culture media that resemble to some extent the conditions prevailing in niches of potential infection. These include the oral cavity, vagina, and the gastrointestinal and urinary tracts. These sites vary in terms of the availability of oxygen, pH (acidic in the vagina and urine, close to neutral in saliva) and various sources of carbon and nitrogen. For *C. albicans*, significant changes in the proteome have often been described for different culture conditions. Previous studies have reported that growth under hypoxic conditions in vagina-simulative medium with iron restriction induces the expression of *C. albicans* typical cell wall proteins Als3, Hwp1, Sim1, Tos1, Utr2, Pir1, Pga10 and Rbt5 [43, 44]. In addition, the pH has a known impact on the cell wall proteome of *C. albicans*; the abundance of different proteins is reported to increase at pH 7.0 (i.e., Als1, Als3, Hyr1, Sod5 and others) compared to pH 4.0 (Phr2, Als4) [45]. Growth media containing different carbon sources (glucose vs. lactate) mediates the plasticity of the fungal cell wall in *C. albicans* cultures and also considerable changes to the composition of the typical cell wall proteins [46].

Using the same method as in this work, i.e., the cell surface shaving with trypsin, proteins present at the surface of various morphological forms of *C. albicans* cells were also characterized [23, 24]. On inspection of this very large group of proteins, a particularly interesting is a large abundance of moonlighting proteins, including Tdh3, Eno1, Pgk1, Adh1, Tal1 and Pdc11 [24]. The presence of such the proteins at the surface of *C. albicans* cells has been explained by their non-classical way of secretion inside the extracellular vesicles [47, 48]. This phenomenon may explain the surface location of not only the above named proteins but also other atypical cell wall proteins of primarily cytoplasmic origin, such as phosphoglycerate mutase (Gpm1) [47], triosephosphate isomerase (Tpi1) and 6-phosphogluconate dehydrogenase (Gnd1) [48].

No detailed data have been reported on changes of cell wall proteome thus far in any species other than *C. albicans*. However, given the changes observed in the epidemiology of infections caused by *Candida* species and, in particular the increase in the incidence of serious candidiases caused by non-albicans *Candida* species such as *C. glabrata*, *C. parapsilosis* and *C. tropicalis* [49], there is an urgent need to analyze the cell wall proteomes of these fungi to devise possible new treatments for these severe infections.

Within the class of typical fungal cell wall proteins that are often involved in wall maintenance, or that can act as fungal virulence factors involved in the adhesion to host cells or hydrolysis of host macromolecular targets, the proteins identified in non-albicans *Candida* species in our present study are potentially very important factors in the infectious diseases caused by these microbes. In *C. glabrata*, the aspartic protease Yps3, shown previously to be required for virulence [50], was detected in cultures grown in DS and AS, the cell wall mannoprotein Cwp1 in AS cultures only, and the glucosidase Scw4/Mp65 in both of these media and in VS. The two latter proteins belong to the core set of proteins consistently present in the cell wall of *C. glabrata* [51] that, together with other typical cell wall proteins with enzymatic activity and adhesins from Epa and Awp family, function in the maintenance and remodeling of the fungal cell wall and adhesion of *C. glabrata* cells [52]. In *C. parapsilosis* and *C. tropicalis*, the cell wall mannoprotein Mp65, the ortholog of which is involved in adhesion and biofilm formation in *C. albicans* [53], was detected after growth in VS and additionally in AS for *C. parapsilosis* and AN for *C. tropicalis*. In addition to these classical cell wall proteins, a significant increase in the surface exposure of moonlighting proteins in different growth media was observed. Some of these proteins were detected at the surface of cultured cells in all of the media tested, such as Eno1 and Tdh3 in *C. glabrata*. These two proteins were detected at the cell surface of all investigated species together with Pdc11. *C. glabrata* Pdc11 was detected in VS, AU and AN cultures, *C. parapsilosis* Eno1, Tdh3 and Pdc11 in DS, AU and AN, *C. tropicalis* Eno1 in DS and AN, and a significant increase in the NSAFs for this protein was observed in AS- and AU-grown cells. *C. tropicalis* Pdc11 and Tdh3 were identified in DS and AN media, whereas an increased level of the latter protein was detected in VS. Moreover, in our previous studies, Pdc11, Eno1 andTdh3 were also identified at the surface of *C. parapsilosis* and *C. tropicalis* cells grown under aerobic conditions in YPD medium, and the latter also in the YPD buffered medium with lowered content of animal-derived peptone (YAPD) [26]. Such the observation strongly emphasizes the widespread presence and abundance of Pdc11, Eno1 and Tdh3 on the cell wall of the investigated species. As demonstrated in other studies, these three proteins located at the cell surfaces of selected non-albicans *Candida* species, additionally including fructose-bisphosphate aldolase (Fba1), Gpm1 and Pgk1 identified also at the cell surface of *C. glabrata* and *C. parapsilosis* in our present analysis, are variously regulated during the response to oxidative stress [17, 18]. Recently, the activity of a transglutaminase was assigned to surface-exposed *C. albicans* Eno1, confirming the important role of this protein in the maintenance of cell wall integrity, in fungal morphological transition and in the protection against osmotic stress [54]. Among the moonlighting proteins identified at the surface of *C. parapsilosis* and *C. tropicalis* in this work, there are also proteins that have been proven to possess strong immunogenic properties. In *C. parapsilosis*, these are proteins such as Eno1, Tdh3, Pdc11, Adh1 and Fba1 [55] and in the case of *C. tropicalis* also Eno1 and Tdh3 [56].

Yeast enolase is a well-known protein that binds human plasminogen [57]. It has been suggested that interactions of *C. albicans* enolase and plasminogen might facilitate the invasion of endothelial cells [58]. This protein was also shown to bind three proteinaceous components of the human plasma contact system—kininogen, coagulation factor XII and plasma prekallikrein—possibly leading to the activation of this system and the generation of the vasoactive and proinflammatory peptides, the kinins [11, 14]. In addition, *C. tropicalis* enolase was identified as a kininogen-binding protein [13]. Some of the proteins identified in our current experiments were suggested previously to be responsible for adhesion to the human extracellular matrix proteins—fibronectin, vitronectin or laminin. This includes *C. parapsilosis* Eno1, Gnd1, phosphoglucose isomerase (Pgi1) and Gpm1 and *C. tropicalis* Eno1 [8]. An ortholog of Tdh3 from the *C. albicans* cell wall was also demonstrated previously to possess fibronectin and laminin binding activity [7].

## Conclusions

The abundant presence of moonlighting proteins at the surfaces of fungal cells under various growth conditions, the observed increases in the levels of these factors under conditions that mimic infectious niches, and the substantial evidence in prior reports for the involvement of many of these particular proteins in pathogen-host interactions, suggest the importance of the functions performed at the cell surface by these molecules in facilitating the adherence of fungal cells to host tissues and the further dissemination of infection. Moreover, the universal occurrence of atypical cell wall proteins with a diversity of physicochemical properties in important fungal pathogens [1] warrants continuing further research on the involvement of these factors in the virulence of these microbes due to their wide impact on human health.

## Methods

### *Candida* strains analyzed in this study and their growth conditions

*C. glabrata* (Anderson) Meyer et Yarrow strain CBS138 (ATCC®2001™), *C. parapsilosis* (Ashford) Langeron et Talice strain CDC 317 (ATCC® MYA-4646™) and *C. tropicalis* (Castellani) Berkhout strain T1 (ATCC® MYA-3404™) were purchased from American Type Culture Collection (Manassas, VA). Cells were cultured in YPD medium (1% yeast extract, 2% soybean peptone and 2% glucose, pH 6.0; Sigma, St. Louis, MO) for 16 h at 30 °C, and 5 x 10^8^ cell aliquots were inoculated into 20 ml of various growth media and further cultured aerobically for 16 h or anaerobically for 72 h at 37 °C on an orbital rotary shaker MaxQ 6000 (170 rpm) (Thermo Fisher Scientific, Waltham, MA). Anoxic conditions were created using a GENbox jar with a GENboxanaer generator (bioMérieux SA, Marcy l’Etoile, France). Cell numbers were determined by optical density measurements at 600 nm.

The compositions of the different growth media used for fungal cultures are as follows: (i) DS - an amino acid-based, chemically defined synthetic medium composed of 5 g/l (NH_4_)_2_SO_4_, 0.2 g/l MgSO_4_∙7H_2_O, 2.5 g/l K_2_HPO_4_,5 g/l NaCl (Avantor Performance Materials Poland S.A., Gliwice, Poland), 0.5 g/l alanine, 1.3 g/l leucine, 1 g/l lysine, 0.1 g/l methionine, 0.5 g/l phenyloalanine, 0.5 g/l proline, 0.5 g/l threonine (BioShop Canada Inc., Burlington, Ontario, Canada), 0.0714 g/l ornithine (Sigma), 0.001 g/l biotin (SERVA Electrophoresis GmbH, Heidelberg, Germany) and 1.25% glucose, pH 6.8, and prepared in strict accordance with the original procedure developed by Lee et al. [35]; (ii) AS – artificial saliva comprising 2.5 g/l mucin from a porcine stomach, type III, 10.0 g/l animal peptone, 5.0 g/l trypticase peptone, 5.0 g/l yeast extract, 5 mg/l hemin, 1 mg/l menadione, 60 mg/l urea (Sigma), 174 mg/l arginine (BioShop) and 2.5 g/l KCl, pH 7.0 [29]; (iii) VS – vagina-simulative medium composed of 18 mg/l of bovine serum albumin (BioShop), 3.5 g/l NaCl, 1.4 g/l KOH, 0.22 g/l Ca(OH)_2_ (Avantor Performance Materials Poland S.A.), 2.2 g/l of 90% lactic acid, 0.32 g/l of 50% glycerol, 0.4 g/l urea (Sigma), 1 g/l of glacial acetic acid (Merck, Darmstadt, Germany) and 0.5% glucose, pH 4.2 [30]; (iv) AU – artificial urine composed of 0.65 g/l CaCl_2_, 0.65 g/l MgCl_2_, 4.6 g/l NaCl, 2.3 g/l Na_2_SO_4_, 0.65 g/l Na_3_C_3_H_5_O(CO_2_)_3_, 0.02 g/l Na_2_C_2_O_4,_ 2.8 g/l KH_2_PO_4_, 1.6 g/l KCl, 1.0 g/l NH_4_Cl (Avantor Performance Materials Poland S.A.), 25 g/l urea, 1.1 g/l of creatinine (Sigma) and 0.3% glucose, pH 5.8 [31]; and (v) AN – anaerobic growth conditions in YPD medium, pH 6.0.

#### Cell surface shaving with trypsin

The release and isolation of peptides from surface-exposed fungal proteins for further identification using shotgun proteomics was performed by cell surface shaving with trypsin as described previously [26] with minor modifications. Briefly, *C. glabrata*, *C. parapsilosis* and *C. tropicalis* cells (5 × 10^8^) cultured under different conditions were harvested by centrifugation (3 min, 3000 rpm) and washed twice with 1 ml of phosphate buffered saline (PBS), pH 7.4 and then twice with 1 ml of 25 mM ammonium bicarbonate buffer (NH_4_HCO_3_). The cells were then mixed with 1 μg of sequencing-grade trypsin (Promega, Madison, WI) in 100 μl of 25 mM NH_4_HCO_3_ and 5 mM dithiothreitol (DTT) (Bioshop). After an incubation for 10 min at 37 °C, cell suspensions were centrifuged (5 min, 3000 rpm), and the peptide-rich supernatants were passed through 0.22 μm filters (Merck) and incubated for an additional 24 h at 37 °C to enhance tryptic digestion. The reaction was subsequently stopped by the addition of trifluoroacetic acid (TFA) (Sigma) to a final concentration of 0.1% and further incubated for 15 min at 4 °C. The protein precipitates were then discarded and the supernatants containing the peptides were centrifuged (12 min, 12,000 rpm) and dried in a Speed-Vac (Martin Christ, Osterode am Harz, Germany). Three independent biological replicates were prepared from the cells of each *Candida* species cultured under particular growth conditions.

#### Protein identification by liquid chromatography-coupled tandem mass spectrometry

The peptides obtained after tryptic digestion of the fungal cultures were identified using high performance liquid chromatography-coupled tandem mass spectrometry (LC-MS/MS). Each peptide-containing sample was dissolved in 110 μl of 10% acetonitrile (ACN) with 0.1% formic acid, centrifuged for 12 min at 12000 rpm) and transferred to new vials. Peptides were separated on a 100 mm × 2.1 mm Aeris 3.6 μm PEPTIDE XB-C18 column (Phenomenex, Torrance, CA), with a 10–55% gradient of 0.1% formic acid in 80% ACN for 60 min via a flow rate of 0.1 ml/min using ultra-high-performance liquid chromatography Dionex Ultimate 3000 system. They were then analyzed using a HCTUltra ETDII ion-trap mass spectrometer equipped with an electrospray ionization ion source and HyStar 3.2 software (Bruker, Bremen, Germany). The mass spectrometer was operated in a standard MS/MS mode with simultaneous fragmentation of the most intensive precursor ions by collision-induced dissociation. The mass range was 200 to 1800 m/z for MS scanning with an Enhanced Mode (speed 8100 m/z/s), the target mass was at 800 m/z and the maximum accumulation time was set to 100 ms to avoid overloading of the ion trap. The data dependent MS/MS scan events were acquired using a smart parameter setting (SPS) with the intensity threshold set at 1 × 10^4^, and a maximum accumulation time of 50 ms. Capillary voltages were set to 4 kV and the capillary temperature was 350 °C.

Mascot Generic format (.mgf) files were generated by pre-processing the raw data with Data Analysis 4.0 software (Bruker). The lists of obtained peaks were searched against the non-redundant NCBI protein database with a taxonomy restriction for fungi (26,490,256 sequences for all entries; 1,924,810 sequences for fungal proteins) using Biotools 3.2 software (Bruker) and an in-house Mascot server v.2.3.0 (Matrix Science, London, UK). The following search parameters were applied: enzyme specificity, trypsin; permitted number of missed cleavages, 2; fixed modification, carbamidomethylation (C); variable modifications, oxidation (M); charge state, 1+, 2+, 3+; C^13^ number, 1; mass values, monoisotopic; experimental peptide mass value tolerance (Mass Tol.) of ±0.3 Da; and a fragment ion mass tolerance (MS/MS Tol.) of ±0.3 Da. After peptide identification, a final dataset was prepared based on the normalized spectral abundance factors (NSAFs) [59] but taking into consideration only those proteins that were identified with a Mascot Score greater than 67 (*p* < 0.05). NSAFs were calculated by dividing the spectral number (SpC) of each protein by its length (L, number of amino acids) and this value was then normalized by dividing by the sum of all SpC/L for all proteins identified in the composite mixture and listed in the Additional file 2: Table S2, Additional file 3: Table S3 and Additional file 4: Table S4 together with the data necessary for the calculation (Additional files 2, 3 and 4).

The statistical significance of the relative change in NSAF values calculated for each cell wall protein identified for a particular growth medium with respect to the defined synthetic medium, was analyzed by the Student *t*-test using GraphPad Prism 6 software (La Jolla, CA).

## Additional files

Additional file 1: **Table S1.** Mass spectrometry identification of *C. glabrata, C. parapsilosis* and *C. tropicalis* proteins present at the cell surface under different growth conditions. (PDF 232 kb)

Additional file 2: **Table S2.** Mass spectrometry identification of *C. glabrata* proteins present at the cell surface under different growth conditions. (PDF 279 kb)

Additional file 3: **Table S3.** Mass spectrometry identification of *C. parapsilosis* proteins present at the cell surface under different growth conditions. (PDF 261 kb)

Additional file 4: **Table S4.** Mass spectrometry identification of *C. tropicalis* proteins present at the cell surface under different growth conditions. (PDF 224 kb)

Additional file 5: **Figure S1.** Relative differences in the level of expression of selected surface-exposed moonlighting proteins depending on the type of medium used. (PDF 171 kb)

## Data Availability

All data generated or analyzed during this study are included in this published article and its supplementary information files.

## Abbreviations

AN: Anaerobic conditions
AS: Artificial saliva
AU: Artificial urine
DS: An amino acid defined synthetic medium
GO: Gene ontology
LC-MS/MS: High performance liquid chromatography-coupled tandem mass spectrometry
NSAFs: Normalized spectral abundance factors
VS: Vagina-simulative medium
