# Supplementary material for: Moonlighting proteins are variably exposed at the cell surfaces of Candida glabrata, Candida parapsilosis and Candida tropicalis under certain growth conditions
Source: BMC Microbiol. 2019 Jul 3;19:149. doi: 10.1186/s12866-019-1524-5 (PMC6609379; doi:10.1186/s12866-019-1524-5)
Supplement: Supplementary file 1 — Table S1. Mass spectrometry identification of C. glabrata, C. parapsilosis and C. tropicalis proteins present at the cell surface under different growth conditions. (PDF 232 kb) [file 12866_2019_1524_MOESM1_ESM.pdf]

Supplementary table 1. Mass spectrometry identification of *C. glabrata, C. parapsilosis* and *C. tropicalis* proteins present at the cell surface under different growth conditions.

Cell surface shaving of fungal cells with trypsin and the additional digestion of the obtained proteins for 24 hours was performed. The resulting peptides were analyzed using the Dionex Ultimate 3000 UHPLC system coupled to an HCTUltra ETDII mass spectrometer. The obtained lists of peaks were searched against the NCBI protein database using an in-house Mascot server. The normalized abundance factors (NSAFs) were calculated for each of the tested growth conditions and the statistical significance with respect to the defined synthetic medium is indicated as follows: **p* values from 0.01 to 0.05; ***p* from 0.001 to 0.01, ****p* from 0.0001 to 0.001;*****p* <0.0001; ns, not significant by Student *t*-test. Molecular function and involvement in similar cellular processes assigned to fungal proteins on the basis of GO Annotations from the *Candida* Genome Database (CGD) and *Saccharomyces* Genome Database (SGD).

C. glabrata									
**NCBI accession number**	**Accession number**	**Protein**	**Description**	**GO annotation - molecular function or biological process**	**defined synthetic medium**	**artificial saliva**	**vagina-simulative medium**	**artificial urine**	**anaerobic conditions**
AAN77243	gi|25992752	pyruvate decarboxylase [*Candida glabrata*]	pyruvate decarboxylase (Pdc1)	pyruvate decarboxylase activity, branched-chain-2-oxoacid decarboxylase activity, magnesium ion binding, thiamine pyrophosphate binding			0.0519645	0.08659	0.0200206
XP_002999583	gi|302309840	60S ribosomal protein L36 [*Candida glabrata* CBS 138]	highly similar to uniprot|O14455 *Saccharomyces cerevisiae* YPL249ca RPL36B	RNA binding, structural constituent of ribosome					0.0197872
XP_449076	gi|302309871	60S ribosomal protein L13 [*Candida glabrata* CBS 138]	highly similar to uniprot|P40212 *Saccharomyces cerevisiae* YMR142c RPL13B	RNA binding, structural constituent of ribosome	0.01418653				0.0290089 ns
XP_444862	gi|50284869	hypothetical protein [*Candida glabrata* CBS 138]	uncharacterized protein CAGL0A02255g	unknown			0.1199572		
XP_444905	gi|50284953	60S acidic ribosomal protein P2 [*Candida glabrata* CBS 138]	highly similar to uniprot|P02400 *Saccharomyces cerevisiae* YDR382w RPLA4	protein kinase activator activity, structural constituent of ribosome	0.04414035				0.0544603 ns
XP_444908	gi|50284959	uncharacterized protein	highly similar to	GTPase activity, GTP		0.011287			0.0109430

		CAGL0A03234g [*Candida glabrata*]	uniprot|P32324 *Saccharomyces cerevisiae* YDR385w elongation factor 2 (Eft2)	binding. rRNA binding, translation elongation factor activity					
XP_445106	gi|50285355	uncharacterized protein CAGL0B03069g [*Candida glabrata*]	highly similar to uniprot|P15019 *Saccharomyces cerevisiae* YLR354c transaldolase (Tal1)	sedoheptulose-7-phosphate:D-glyceraldehyde-3-phosphate glyceronetransferase activity, carbohydrate metabolic process, pentose-phosphate shunt	0.0186313	0.03913*			0.0162104 ns
XP_445155	gi|50285453	60S ribosomal protein L4 [*Candida glabrata*]	highly similar to uniprot|P10664 *Saccharomyces cerevisiae* YBR031w RPL2A	RNA binding, structural constituent of ribosome	0.0254646				0.0157310 ns
XP_445189	gi|50285521	uncharacterized protein CAGL0C00110g [*Candida glabrata*]	some similarities with uniprot|P36170 *Saccharomyces cerevisiae* YKR102w flocculation protein (Flo10)	mannose binding			0.0367593		
XP_445408	gi|50285959	uncharacterized protein CAGL0C05379g [*Candida glabrata*]	highly similar to uniprot|P40150 *Saccharomyces cerevisiae* YNL209w ribosome-associated molecular chaperone (Ssb2)	ATPase activity, ATP binding, unfolded protein binding, cytoplasmic translation	0.0207243			0.058375*	0.0322794**
XP_445466	gi|50286075	uncharacterized protein CAGL0D01188g [*Candida glabrata*]	highly similar to uniprot|P02994 *Saccharomyces cerevisiae* YPR080w elongation factor 1-alpha (Tef1)	ribosome binding, translation elongation factor activity,	0.01886806			0.041569*	
XP_445471	gi|50286085	uncharacterized protein CAGL0D01298g [*Candida glabrata*]	highly similar to uniprot|P23254 *Saccharomyces cerevisiae* YPR074c transketolase (Tkl1)	metal ion binding, transketolase activity		0.018662			0.0139828
XP_445725	gi|50286593	uncharacterized protein CAGL0E00869g [*Candida glabrata*]	similar to uniprot|P39015 *Saccharomyces cerevisiae* YLR150w suppressor protein (Mtp4)	DNA binding, ribosome binding, telomeric DNA binding, triplex DNA binding, translation		0.034394			
XP_445764	gi|50286669	uncharacterized protein CAGL0E01727g	similar to uniprot|P32329 *Saccharomyces cerevisiae*	aspartic-type endopeptidase activity, fungal-type cell	0.0230774	0.029228 ns			

		[Candida glabrata]	YLR120c aspartic proteinase 3 (Yap3)	wall organization					
XP_445776	gi|50286693	40S ribosomal protein S19 [*Candida glabrata*]	highly similar to uniprot|P07280 *Saccharomyces cerevisiae* YOL121c RP55A	structural constituent of ribosome	0.0508370			0.088625 ns	
XP_445858	gi|50286857	60S ribosomal protein L8 [*Candida glabrata*]	highly similar to uniprot|P29453 *Saccharomyces cerevisiae* YLL045c RPL4B	RNA binding, structural constituent of ribosome				0.02993	
XP_445966	gi|50287073	uncharacterized protein CAGL0E06358g [*Candida glabrata*]	highly similar to uniprot|P00950 *Saccharomyces cerevisiae* YKL152c phosphoglycerate mutase 1 (Gpm1)	2,3-bisphosphoglycerate-dependent phosphoglycerate mutase activity, phosphoglycerate mutase activity, gluconeogenesis, glycolytic process	0.0287052				0.0275597 ns
XP_446298	gi|50287737	uncharacterized protein CAGL0F07601g [*Candida glabrata*]	similar to uniprot|P28319 *Saccharomyces cerevisiae* YKL096w cell wall mannoprotein (Cwp1)	structural constituent of cell wall		0.046101			
XP_446378	gi|50287897	uncharacterized protein CAGL0G00308g [*Candida glabrata*]	similar to uniprot|P53334 *Saccharomyces cerevisiae* YGR279c probable family 17 glucosidase (Scw/Mp65)	glucosidase activity, carbohydrate metabolic process, cell wall organization	0.0204128	0.054188 ns	0.1054126***		
XP_446529	gi|50288201	uncharacterized protein CAGL0G03795g [*Candida glabrata*]	highly similar to uniprot|P10592 *Saccharomyces cerevisiae* YLL024c heat shock protein (Ssa2)	ATPase activity, ATP binding, tRNA binding, unfolded protein binding	0.01571147				0.0178035 ns
XP_446644	gi|50288429	40S ribosomal protein S7 [*Candida glabrata*]	highly similar to uniprot|P26786 *Saccharomyces cerevisiae* YOR096w RP30	structural constituent of ribosome, cytoplasmic translation	0.02650804			0.039844*	
XP_446675	gi|50288491	40S ribosomal protein S18 [*Candida glabrata*]	highly similar to uniprot|P35271 *Saccharomyces cerevisiae* YML026c RPS18EB	structural constituent of ribosome, cytoplasmic translation				0.075769	0.0430385
XP_446770	gi|50288681	uncharacterized protein CAGL0G09383g [*Candida glabrata*]	highly similar to uniprot|P00359 *Saccharomyces cerevisiae* YGR192c glyceraldehyde-3-phosphate dehydrogenase	glyceraldehyde-3-phosphate dehydrogenase (NAD+) (phosphorylating) activity, gluconeogenesis, glycolytic process	0.1020257	0.066536 ns	0.2637412*	0.107388 ns	0.0861923 ns

			3 (Tdh3)						
XP_446818	gi|50288777	40S ribosomal protein S5 [*Candida glabrata*]	highly similar to uniprot|P26783 *Saccharomyces cerevisiae* YJR123w RPS5	mRNA binding, rRNA binding, structural constituent of ribosome, cytoplasmic translation	0.0154632			0.043095**	
XP_447033	gi|50289205	uncharacterized protein CAGL0H05445g [*Candida glabrata*]	highly similar to uniprot|P12709 Saccharomyces cerevisiae YBR196c glucose-6-phosphate isomerase (Pgi1)	glucose-6-phosphate isomerase activity, gluconeogenesis, glycolytic process, pentose-phosphate shunt		0.023119			
XP_447041	gi|50289221	40S ribosomal protein S6 [*Candida glabrata*]	highly similar to uniprot|P02365 *Saccharomyces cerevisiae* YPL090c RPS10B	structural constituent of ribosome, cytoplasmic translation				0.031857	0.0364656
XP_447084	gi|50289307	uncharacterized protein CAGL0H06633g [*Candida glabrata*]	highly similar to uniprot|P10963 *Saccharomyces cerevisiae* YKR097w phosphoenolpyruvate carboxykinase (Pck1)	ATP binding, phosphoenolpyruvate carboxykinase (ATP) activity, gluconeogenesis				0.038839	
XP_447161	gi|50289459	uncharacterized protein CAGL0H08327g [*Candida glabrata*]	highly similar to uniprot|P00942 *Saccharomyces cerevisiae* YDR050c triosephosphate isomerase (Tpi1)	triose-phosphate isomerase activity, gluconeogenesis, glycolytic process		0.070534			0.0377085
XP_447227	gi|50289591	uncharacterized protein CAGL0H09878g [*Candida glabrata*]	highly similar to uniprot|P00817 *Saccharomyces cerevisiae* YBR011c inorganic pirophosphatase (Ipp1)	inorganic diphosphatase activity, magnesium ion binding					0.0377302
XP_447360	gi|50289857	uncharacterized protein CAGL0I02486g [*Candida glabrata*]	highly similar to uniprot|P00924 *Saccharomyces cerevisiae* YGR254w enolase I (Eno1)	magnesium ion binding, phosphopyruvate hydratase activity, gluconeogenesis, glycolytic process	0.0489215	0.088689*	0.1891493 ns	0.081577*	0.1263368*
XP_447438	gi|50290013	uncharacterized protein CAGL0I04356g [*Candida glabrata*]	highly similar to uniprot|P10081 *Saccharomyces cerevisiae* YKR059w translation initiation factor eIF4A (Tif1)	ATP binding, ATP-dependent RNA helicase activity, RNA-dependent ATPase activity, translation initiation factor activity		0.028099			
XP_447518	gi|50290173	uncharacterized protein CAGL0I06160g [*Candida*	highly similar to uniprot|P47001	structural constituent of cell wall		0.034297			

		glabrata]	*Saccharomyces cerevisiae* YJL158c cell wall mannoprotein (Cis3)						
XP_447590	gi|50290317	uncharacterized protein CAGL0I07843g [*Candida glabrata*]	highly similar to uniprot|P00330 *Saccharomyces cerevisiae* YOL086c alcohol dehydrogenase I (Adh1)	alcohol dehydrogenase (NAD) activity, ethanol biosynthetic process involved in glucose fermentation to ethanol				0.04322	0.0385497
XP_447888	gi|50290911	uncharacterized protein CAGL0J04202g [*Candida glabrata*]	similar to uniprot|P22943 *Saccharomyces cerevisiae* YFL014w heat shock protein (Hsp12)	lipid binding, plasma membrane organization	0.0861709				0.0276680 ns
XP_447969	gi|50291073	uncharacterized protein CAGL0J06050g [*Candida glabrata*]	similar to uniprot|P38616 *Saccharomyces cerevisiae* YNL160w secreted glycoprotein (Ygp1)	asparagine catabolic process, cell wall assembly	0.0723081	0.070267 ns	0.1912018 ns		
XP_448200	gi|50291535	40S ribosomal protein S3 [*Candida glabrata*]	highly similar to uniprot|P05750 *Saccharomyces cerevisiae* YNL178w RPS3	structural constituent of ribosome, cytoplasmic translation		0.050979		0.045367	0.0240331
XP_448368	gi|50291871	uncharacterized protein CAGL0K03289g [*Candida glabrata*]	highly similar to uniprot|Q03161 *Saccharomyces cerevisiae* YMR099c glucose-6-phosphate 1-epimerase (Gpe1)	carbohydrate binding, glucose-6-phosphate 1-epimerase activity					0.0078718
XP_448490	gi|50292115	40S ribosomal protein S17 [*Candida glabrata*]	highly similar to uniprot|P14127 *Saccharomyces cerevisiae* YDR447c RP51B	structural constituent of ribosome, cytoplasmic translation				0.027539	
XP_448549	gi|50292233	uncharacterized protein CAGL0K07546g [*Candida glabrata*]	similar to uniprot|P36069 *Saccharomyces cerevisiae* YKL128c suppressor of TPS2 mutant, probable phosphoglycerate mutase (Pmu1)	isomerase activity			0.3150439		
XP_448719	gi|50292573	uncharacterized protein CAGL0K11572g [*Candida glabrata*]	similar to uniprot|P28707 *Saccharomyces cerevisiae* YKL117w Hsp90 Associated Co-chaperone (Sba1)	chaperone binding					0.0090767
XP_448731	gi|50292597	uncharacterized protein CAGL0K11858g	highly similar to uniprot|Q12335	FMN binding, identical protein binding, NAD(P)H		0.111995			0.0411690

		[*Candida glabrata*]	*Saccharomyces cerevisiae* YDR032c protoplast secreted protein 2 (Pst2)	dehydrogenase (quinone) activity					
XP_448795	gi|50292725	uncharacterized protein CAGL0L00495g [*Candida glabrata*]	highly similar to uniprot|P15108 *Saccharomyces cerevisiae* YMR186w heat shock protein (Hsc82)	ATPase activity, unfolded protein binding, protein folding	0.0233118	0.05873*			0.0163857 ns
XP_448802	gi|50292739	acetate-CoA ligase [*Candida glabrata*]	highly similar to uniprot|Q01574 *Saccharomyces cerevisiae* YAL054c acetyl-CoA synthetase (Acs1)	acetate-CoA ligase activity, acetate fermentation				0.013773	
XP_448879	gi|50292893	uncharacterized protein CAGL0L02497g [*Candida glabrata*]	highly similar to uniprot|P14540 *Saccharomyces cerevisiae* YKL060c fructose-bisphosphate aldolase (Fba1)	fructose-bisphosphate aldolase activity, gluconeogenesis, glycolytic process	0.0114918				0.0444462*
XP_449113	gi|50293403	uncharacterized protein CAGL0L07722g [*Candida glabrata*]	highly similar to uniprot|P00560 *Saccharomyces cerevisiae* YCR012w phosphoglycerate kinase (Pgk1)	ATP binding, phosphoglycerate kinase activity, gluconeogenesis, glycolytic process	0.0351108	0.030844 ns		0.036836 ns	0.0559054 ns
XP_449292	gi|50293761	uncharacterized protein CAGL0L12056g [*Candida glabrata*]	highly similar to uniprot|P29311 *Saccharomyces cerevisiae* YER177w protein involved in rapamycin-sensitive signalling	DNA replication origin binding, phosphoserine residue binding, protein domain specific binding, RNA polymerase II activating transcription factor binding		0.063676			
XP_449352	gi|50293881	uncharacterized protein CAGL0M00176g [*Candida glabrata*]	highly similar to uniprot|P47176 *Saccharomyces cerevisiae* YJR148w branched-chain-amino-acid aminotransferase, cytosolic (Twt2)	branched-chain-amino-acid transaminase activity, L-isoleucine transaminase activity, L-leucine transaminase activity, L-valine transaminase activity		0.017077			
XP_449381	gi|50293939	40S ribosomal protein S1 [*Candida glabrata*]	highly similar to uniprot|P33442 *Saccharomyces cerevisiae* YLR441c RP10A	structural constituent of ribosome, cytoplasmic translation	0.0166065			0.04507****	

XP_449464	gi|50294105	60S ribosomal protein L5 [*Candida glabrata*]	highly similar to uniprot|P26321 *Saccharomyces cerevisiae* YPL131w RPL1	5S rRNA binding, structural constituent of ribosome	0.0261358				0.0161565 ns
XP_449850	gi|50294878	uncharacterized protein CAGL0M11704g [*Candida glabrata*]	similar to uniprot|P38013 *Saccharomyces cerevisiae* YLR109w alkyl hydroperoxide reductase (Ahp1)	thioredoxin peroxidase activity, cell redox homeostasis, cellular response to oxidative stress					0.0315610
XP_449865	gi|50294908	uncharacterized protein CAGL0M12034g [*Candida glabrata*]	highly similar to uniprot|P00549 *Saccharomyces cerevisiae* YAL038w pyruvate kinase (Cdc19)	pyruvate kinase activity, glycolytic process, pyruvate metabolic process	0.0142592			0.053527****	0.0315165*
XP_449923	gi|50295024	uncharacterized protein CAGL0M13343g [*Candida glabrata*]	highly similar to uniprot|P38720 *Saccharomyces cerevisiae* YHR183w 6-phosphogluconate dehydrogenase (Gnd1)	phosphogluconate dehydrogenase (decarboxylating) activity, cellular response to oxidative stress, D-gluconate metabolic process, pentose-phosphate shunt, oxidative branch	0.0073046			0.014145 ns	
AAF81925	gi|8927040	elongation factor 2, partial [*Candida glabrata*]	elongation factor 2 (Eft2)	GTPase activity, translation elongation factor activity, maintenance of translational fidelity, positive regulation of translational elongation, translational elongation	0.0105131			0.039102***	
C. parapsilosis									
**NCBI accession number**	**Accession number**	**Protein**	**Description**	**GO annotation - molecular function or biological process**	**defined synthetic medium**	**artificial saliva**	**vagina-simulative medium**	**artificial urine**	**anaerobic conditions**
CCE39876	gi|354543158	hypothetical protein CPAR2_602950 [*Candida parapsilosis*]	phosphoglycerate kinase (Pgk1)	phosphoglycerate kinase activity; gluconeogenesis, glycolytic process,induction by				0.126005	0.08621

				symbiont of host defense response					
CCE39895	gi|354543177	hypothetical protein CPAR2_603140 [*Candida parapsilosis*]	putative ketol-acid reductoisomerase (Ilv5)	coenzyme binding; double-stranded DNA binding; ketol-acid reductoisomerase activity					0.0178176
CCE40124	gi|354543405	hypothetical protein CPAR2_101620 [*Candida parapsilosis*]	acetyl-coA hydrolase (Ach1)	acetate CoA-transferase activity, acetyl-CoA hydrolase activity, succinyl-CoA hydrolase activity, acetate metabolic process					0.0102072
CCE40129	gi|354543410	hypothetical protein CPAR2_101670 [*Candida parapsilosis*]	heat shock protein (Hsp12)	response to stress in cell wall					0.0541540
CCE40514	gi|354543792	hypothetical protein CPAR2_105500 [*Candida parapsilosis*]	eisosome component (Pil1)	lipid binding, eisosome assembly, endocytosis, negative regulation of protein kinase activity, response to heat					0.0252469
CCE40659	gi|354543937	hypothetical protein CPAR2_106940 [*Candida parapsilosis*]	heat shock protein (Ssa2)	ATP binding, heat shock protein binding, peptide binding, tRNA binding, unfolded protein binding	0.0376708	0.0547124 ns		0.0496908 ns	0.0705495 ns
CCE40698	gi|354543976	hypothetical protein CPAR2_107330 [*Candida parapsilosis*]	orf19.2460	unknown		0.177987	0.13888579		
CCE40863	gi|354544140	hypothetical protein CPAR2_109010 [*Candida parapsilosis*]	orf19.3053	unknown in biofilm matrix					0.0211803
CCE40929	gi|354544206	hypothetical protein CPAR2_109660 [*Candida parapsilosis*]	pH-responsive protein 2 (Phr2)	1,3-beta-glucanosyltransferase activity, cellular response to pH, chromatin silencing, filamentous growth, fungal-type cell wall (1->3)-beta-D-glucan biosynthetic process, fungal-type cell wall organization, pathogenesis,	0.017018	0.03967***			
CCE41227	gi|354544503	hypothetical protein CPAR2_302160 [*Candida parapsilosis*]	ribosomal protein A6	structural constituent of ribosome (IEA with S. cerevisiae: RPS6A)				0.09032023	

CCE41277	gi|354544553	hypothetical protein CPAR2_302650 [*Candida parapsilosis*]	beta subunit of fatty acid synthetase (Fas1)	3-hydroxyacyl-[acyl-carrier-protein] dehydratase activity, cellular response to drug, long-chain fatty acid biosynthetic process, oxidation-reduction process,					0.0040278
CCE41925	gi|354545198	hypothetical protein CPAR2_804740 [*Candida parapsilosis]*	transketolase(Tkl1)	transketolase activity, pentose-phosphate shunt,					0.0181811
CCE42024	gi|354545297	hypothetical protein CPAR2_805730 [*Candida parapsilosis*]	orf19.2168.3	ER-dependent peroxisome organization, endoplasmic reticulum inheritance, endoplasmic reticulum tubular network membrane organization, nuclear pore complex assembly, response to endoplasmic reticulum stress, vesicle-mediated transport,					0.0149871
CCE42100	gi|354545372	hypothetical protein CPAR2_806490 [*Candida parapsilosis*]	cell wall protein (Pir1)	structural constituent of cell wall, cellular response to temperature stimulus, fungal-type cell wall organization, intracellular protein transport,	0.0532944	0.102017**	0.11587458*		
CCE42118	gi|354545390	hypothetical protein CPAR2_806670 [*Candida parapsilosis*]	yeast wall protein (Ywp1)	adhesion of symbiont to host, single-species biofilm formation	0.0247977	0.048285 ns	0.0842443*	0.0434768*	
CCE42249	gi|354545521	hypothetical protein CPAR2_807980 [*Candida parapsilosis*]	triosephosphate isomerase (Tpi)	canonical glycolysis, cellular response to starvation, filamentous growth of a population of unicellular organisms in response to biotic stimulus, filamentous growth of a population of unicellular organisms in response to starvation, induction by symbiont of host defense response,					0.040401
CCE42318	gi|354545590	hypothetical protein CPAR2_808670 [*Candida*	glyceraldehyde-3-phosphate	NAD binding, NADP binding, glyceraldehyde-3-	0.0716658			0.1154571 ns	0.0748528 ns

		parapsilosis]	dehydrogenase(Tdh3)	phosphate dehydrogenase (NAD+) (phosphorylating) activity, glucose metabolic process, heme transport, induction by symbiont of host defense response, oxidation-reduction process, reactive oxygen species metabolic process					
CCE42494	gi|354545766	hypothetical protein CPAR2_201370 [*Candida parapsilosis*]	similar to ASR2, gene regulated by cAMP and by osmotic stress	unknown					0.01933424
CCE42617	gi|354545888	hypothetical protein CPAR2_202600 [*Candida parapsilosis*]	transaldolase (Tal1)	sedoheptulose-7-phosphate:D-glyceraldehyde-3-phosphate glyceronetransferase activity, carbohydrate metabolic process, pentose-phosphate shunt	0.0663434			0.0849522 ns	0.0200083**
CCE42709	gi|354545980	hypothetical protein CPAR2_203520 [*Candida parapsilosis*]	6-phosphogluconate dehydrogenase (Gnd1)	NADP binding, phosphogluconate dehydrogenase (decarboxylating) activity, D-gluconate catabolic process, cellular response to oxidative stress, glycolytic process via Entner-Doudoroff Pathway, oxidation-reduction process, pentose-phosphate shunt, oxidative branch,					0.0105731
CCE42845	gi|354546116	hypothetical protein CPAR2_204880 [*Candida parapsilosis*]	phosphoglucose isomerase (Pgi1)	glucose-6-phosphate isomerase activity, gluconeogenesis, glycolytic process, pentose-phosphate shunt					0.0112716
CCE43078	gi|354546348	hypothetical protein CPAR2_207210 [*Candida parapsilosis*]	enolase (Eno1)	phosphopyruvate hydratase activity, protein-glutamine gamma-glutamyltransferase activity, filamentous growth of a population of unicellular organisms in	0.072136			0.0950709 ns	0.0657891 ns

				response to biotic stimulus, fungal-type cell wall organization or biogenesis, glycolytic process, induction by symbiont of host defense response,					
CCE43208	gi|354546478	hypothetical protein CPAR2_208530 [*Candida parapsilosis*]	putative inducible acid phosphatase (Pho100)	hydrolase activity, pathogenesis,			0.14202434		
CCE43537	gi|354546805	hypothetical protein CPAR2_211810 [*Candida parapsilosis*]	phosphoglycerate mutase (Gpm1)	2,3-bisphosphoglycerate-dependent phosphoglycerate mutase activity, gluconeogenesis, glycolytic process, interaction with host					0.0705204
CCE43577	gi|354546845	hypothetical protein CPAR2_212210 [*Candida parapsilosis*]	NAD-aldehyde dehydrogenase (Ald5)	aldehyde dehydrogenase (NAD) activity, acetate biosynthetic process		0.2342838			
CCE43824	gi|354547091	hypothetical protein CPAR2_500500 [*Candida parapsilosis*]	orf19.1239	unknown, secreted protein		0.056851			
CCE43876	gi|354547143	hypothetical protein CPAR2_501020 [*Candida parapsilosis*]	pyruvate decarboxylase (Pdc11)	branched-chain-2-oxoacid decarboxylase activity, magnesium ion binding, pyruvate decarboxylase activity, thiamine pyrophosphate binding	0.019848			0.0327859***	0.0297271 ns
CCE43989	gi|354547255	hypothetical protein CPAR2_502140 [*Candida parapsilosis*]	chitinase 2 (Cht2)	catalytic activity, hydrolase activity, hydrolyzing O-glycosyl compounds,carbohydrate metabolic process cellular response to starvation, filamentous growth of a population of unicellular organisms in response to starvation (IEA with C. albicans: CHT2)	0.0340471	0.107761*	0.0526054 ns		
CCE44033	gi|354547299	hypothetical protein CPAR2_502580 [*Candida parapsilosis*]	alcohol dehydrogenase (Adh1)	carbonyl reductase (NADPH) activity, alcohol dehydrogenase (NAD) activity, methylglyoxal reductase (NADH-		0.13295892		0.0836480	0.1399144

				dependent) activity, zinc ion binding, ethanol biosynthetic process involved in glucose fermentation to ethanol, ethanol catabolic process					
CCE44070	gi|354547336	hypothetical protein CPAR2_502950 [*Candida parapsilosis*]	plasma membrane H(+)-ATPase (Pma1)	proton binding, proton-exporting ATPase activity, phosphorylative mechanism					0.0059106
CCE44321	gi|354547586	hypothetical protein CPAR2_401230 [*Candida parapsilosis*]	fructose-bisphosphate aldolase (Fba1)	fructose-bisphosphate aldolase activity, zinc ion binding, aspartate transmembrane transport, gluconeogenesis, glycolytic process, induction by symbiont of host defense response					0.0426764
CCE44680	gi|354547945	hypothetical protein CPAR2_404840 [*Candida parapsilosis*]	peroxiredoxine (Tsa1)	thioredoxin peroxidase activity, cell redox homeostasis, cellular detoxification of hydrogen peroxide, chaperone-mediated protein folding					0.0271258
CCE44939	gi|354548203	hypothetical protein CPAR2_407410 [*Candida parapsilosis*]	cell wall mannoprotein (Mp65)	catalytic activity, hydrolase activity, hydrolyzing O-glycosyl compounds, carbohydrate metabolic process, cell adhesion involved in single-species biofilm formation, cellular response to glucose starvation, cellular response to neutral pH, conjugation with cellular fusion, induction by symbiont of host defense response, pathogenesis	0.0610849	0.114698 ns	0.3037744 ns		
CCE45034	gi|354548298	hypothetical protein CPAR2_700380 [*Candida parapsilosis*]	heat shock protein (Hsp70)	peptide binding, unfolded protein binding, SRP-dependent cotranslational protein targeting to membrane, translocation,					0.0456689

				cellular response to heat, entry into host cell, induction by symbiont of host defense response, protein folding, response to toxic substance					
CCE45261	gi|354548524	hypothetical protein CPAR2_702740 [*Candida parapsilosis*]	peptidyl-prolyl cis-trans isomerase (Cyp1)	cyclosporin A binding, peptidyl-prolyl cis-trans isomerase activity, ascospore formation, histone deacetylation, positive regulation of meiotic nuclear division, protein folding, protein peptidyl-prolyl isomerization					0.0472610
CCE45302	gi|354548565	hypothetical protein CPAR2_703150 [*Candida parapsilosis*]	heat shock protein (Hsc82)	ATP binding, ATPase activity, coupled, chaperone binding, unfolded protein binding	0.0257079				0.0476734 ns
AF107291_1	gi|8927048	elongation factor 2, partial, protein CPAR2_211630 [*Candida parapsilosis*]	elongation factor 2 (Eft2)	GTP binding, GTPase activity, drug binding, translation elongation factor activity					0.00531587
				C. tropicalis					
**NCBI Accession number**	**Accession number**	**Protein**	**Description**	**GO annotation - molecular function or biological process**	**defined synthetic medium**	**artificial saliva**	**vagina-simulative medium**	**artificial urine**	**anaerobic conditions**
XP_002545696	gi|255721523	predicted protein CTRG_00477 [*Candida tropicalis* MYA-3404]	cell wall protein (Rbt1)	fungal-type cell wall organization, pathogenesis		0.180324			0.040989
XP_002546108	gi|255722347	hypothetical protein CTRG_00890 [*Candida tropicalis* MYA-3404]	putative constitutive acid phosphatase (Pho113)	acid phosphatase activity			0.246955		
XP_002546341	gi|255722814	thioredoxin II CTRG_05819 [*Candida tropicalis* MYA-3404]	thioredoxin (Trx)	protein disulfide oxidoreductase activity, antioxidant activity, electron transfer activity,					0.290615

				protein disulfide oxidoreductase activity					
XP_002546924	gi|255723990	hypothetical protein CTRG_01230 [*Candida tropicalis* MYA-3404]	phospholipase B (Plb)	phospholipase activity, phospholipid catabolic process					0.016093
XP_002547549	gi|255725240	predicted protein CTRG_01856 [*Candida tropicalis MYA-3404]*	cell wall protein (Ywp1)	adhesion of symbiont to host, single-species biofilm formation	0.006832				0.030173*
XP_002547786	gi|255725714	hypothetical protein CTRG_02093 [*Candida tropicalis* MYA-3404]	cell wall mannoprotein (Mp65)	catalytic activity, hydrolase activity, hydrolyzing O-glycosyl compounds, carbohydrate metabolic process, cell adhesion involved in single-species biofilm formation, cellular response to glucose starvation, cellular response to neutral pH, conjugation with cellular fusion, induction by symbiont of host defense response, pathogenesis	0.028732		0.186120** **		0.148335*
XP_002548866	gi|255727881	enolase 1 [CTRG_03163 *Candida tropicalis* MYA-3404]	enolase 1 (Eno1)	phosphopyruvate hydratase activity, protein-glutamine gamma-glutamyltransferase activity, filamentous growth of a population of unicellular organisms in response to biotic stimulus, fungal-type cell wall organization or biogenesis, glycolytic process, induction by symbiont of host defense response,	0.088115	0.270533**		0.3236277**	0.159336 ns
XP_002549287	gi|255728723	opaque-phase-specific protein OP4 precursor CTRG_03584 [*Candida tropicalis* MYA-3404]	secreted Ala- Leu- and Ser-rich protein (Op4)	unknown		0.321336			
XP_002549529	gi|255729208	pyruvate decarboxylase CTRG_03826 [*Candida tropicalis* MYA-3404]	pyruvate decarboxylase (Pdc11)	branched-chain-2-oxoacid decarboxylase activity, magnesium ion binding, pyruvate decarboxylase activity, thiamine	0.043406				0.037222 ns

				pyrophosphate binding					
XP_002549835	gi|255729820	conserved hypothetical protein CTRG_04132 [*Candida tropicalis* MYA-3404]	putative inducible acid phosphatase (Pho100)	acid phosphatase activity, pathogenesis	0.033161				0.074489****
XP_002549999	gi|255730149	protein EPD1 precursor CTRG_04296 [*Candida tropicalis* MYA-3404]	pH-responsive protein 2 (Phr2)	1,3-beta-glucanosyltransferase activity, cellular response to pH, chromatin silencing, filamentous growth, fungal-type cell wall (1->3)-beta-D-glucan biosynthetic process, fungal-type cell wall organization, pathogenesis,					0.037634
XP_002550795	gi|255731742	FK506-binding protein CTRG_05093 [*Candida tropicalis* MYA-3404]	FKBP-type peptidyl-prolyl cis-trans isomerase (Rbp1)	drug binding, peptidyl-prolyl cis-trans isomerase activity, macrolide binding					0.148451
XP_002551368	gi|255732890	glyceraldehyde-3-phosphate dehydrogenase CTRG_05666 [*Candida tropicalis* MYA-3404]	glyceraldehyde-3-phosphate dehydrogenase (Tdh3)	NAD binding, NADP binding, glyceraldehyde-3-phosphate dehydrogenase (NAD+) (phosphorylating) activity, glucose metabolic process, heme transport, induction by symbiont of host defense response, oxidation-reduction process, reactive oxygen species metabolic process	0.097361		0.577328****		0.095335 ns
