# Supplementary material for: Moonlighting proteins are variably exposed at the cell surfaces of Candida glabrata, Candida parapsilosis and Candida tropicalis under certain growth conditions
Source: BMC Microbiol. 2019 Jul 3;19:149. doi: 10.1186/s12866-019-1524-5 (PMC6609379; doi:10.1186/s12866-019-1524-5)
Supplement: Supplementary file 2 — Table S2. Mass spectrometry identification of C. glabrata proteins present at the cell surface under different growth conditions. (PDF 279 kb) [file 12866_2019_1524_MOESM2_ESM.pdf]

Supplementary table 2. Mass spectrometry identification of *C. glabrata* proteins present at the cell surface under different growth conditions.

Cell surface shaving of fungal cells with trypsin and the additional digestion of the obtained proteins for 24 hours was performed. The resulting peptides were analyzed using the Dionex Ultimate 3000 UHPLC system coupled to an HCTUltra ETDII mass spectrometer. The obtained lists of peaks were searched against the NCBI protein database using an in-house Mascot server.

Accession number	Protein	Molecular mass [Da]	Number of amino acids	Score	Matches	Sequences	Sequence coverage [%]
defined synthetic medium (DS) 1							
gi|50289857	hypothetical protein [*Candida glabrata* CBS 138]	46767	437	821	17	12	32
gi|50288681	hypothetical protein [*Candida glabrata* CBS 138]	35985	332	734	15	12	48
gi|50292725	hypothetical protein [*Candida glabrata* CBS 138]	80983	705	444	11	10	21
gi|50294560	hypothetical protein [*Candida glabrata* CBS 138]	62152	554	429	17	12	39
gi|50285959	hypothetical protein [*Candida glabrata* CBS 138]	66470	613	410	9	8	19
gi|50288201	hypothetical protein [*Candida glabrata* CBS 138]	69640	640	393	9	9	24
gi|50291073	hypothetical protein [*Candida glabrata* CBS 138]	37779	368	329	13	9	35
gi|50285453	60S ribosomal protein L4 [*Candida glabrata* CBS 138]	39009	362	290	7	7	26
gi|50293403	hypothetical protein [*Candida glabrata* CBS 138]	44704	416	274	8	8	25
gi|50294908	hypothetical protein [*Candida glabrata* CBS 138]	54975	501	271	6	6	16
gi|50295024	hypothetical protein [*Candida glabrata* CBS 138]	53878	489	248	3	3	11

gi|8927040	elongation factor 2 [*Candida glabrata*]	90394	814	235	6	6	11
gi|50286075	hypothetical protein [*Candida glabrata* CBS 138]	50160	458	219	8	7	26
gi|50290911	hypothetical protein [*Candida glabrata* CBS 138]	11173	103	200	7	5	79
gi|50287073	hypothetical protein [*Candida glabrata* CBS 138]	27574	297	190	5	5	25
gi|50293967	hypothetical protein [*Candida glabrata* CBS 138]	34778	324	170	6	4	15
gi|50287897	hypothetical protein [*Candida glabrata* CBS 138]	39767	374	164	4	3	11
gi|50286693	40S ribosomal protein S19 [*Candida glabrata* CBS 138]	15838	144	136	3	2	18
gi|50288781	hypothetical protein [*Candida glabrata* CBS 138]	54176	507	133	5	5	14
gi|50290317	hypothetical protein [*Candida glabrata* CBS 138]	37920	352	132	4	4	12
gi|50288429	40S ribosomal protein S7 [*Candida glabrata* CBS 138]	21531	188	132	4	4	28
gi|50286621	hypothetical protein [*Candida glabrata* CBS 138]	17495	162	127	6	4	38
gi|50293939	40S ribosomal protein S1 [*Candida glabrata* CBS 138]	28770	255	126	3	3	16
gi|302309871	60S ribosomal protein L13 [*Candida glabrata* CBS 138]	22608	199	125	2	2	11
gi|50288123	hypothetical protein [*Candida glabrata* CBS 138]	35129	311	121	5	5	27
gi|50288777	40S ribosomal protein S5 [*Candida glabrata* CBS 138]	25194	226	113	2	2	15
gi|50291535	40S ribosomal protein S3 [*Candida glabrata* CBS 138]	27189	347	112	3	3	26
gi|50294762	60S ribosomal protein L14 [*Candida glabrata* CBS 138]	15238	137	110	3	3	31
gi|50285355	hypothetical protein [*Candida glabrata* CBS 138]	36811	334	100	3	3	14

gi|50292739	acetate--CoA ligase [*Candida glabrata* CBS 138]	79670	704	96	2	2	5
gi|50286231	hypothetical protein [*Candida glabrata* CBS 138]	73342	677	95	2	2	3
gi|302309840	60S ribosomal protein L36 [*Candida glabrata* CBS 138]	11044	100	90	2	2	22
gi|49617210	epithelial adhesin 6 [*Candida glabrata*]	78587	715	89	2	2	2
gi|50294105	60S ribosomal protein L5 [*Candida glabrata* CBS 138]	33741	297	86	3	2	13
gi|50287337	60S ribosomal protein L12 [*Candida glabrata* CBS 138]	17799	165	86	2	2	18
gi|50285685	hypothetical protein [*Candida glabrata* CBS 138]	49787	449	83	2	2	6
gi|50290013	hypothetical protein [*Candida glabrata* CBS 138]	44930	396	79	2	1	5
gi|50292893	hypothetical protein [*Candida glabrata* CBS 138]	39503	361	79	2	2	5
gi|3786314	translation elongation factor3 [*Candida glabrata*]	116793	1045	78	2	2	2
gi|50284953	60S acidic ribosomal protein P2 [*Candida glabrata* CBS 138]	10954	109	74	2	2	22
gi|50285071	40S ribosomal protein S8 [*Candida glabrata* CBS 138]	22540	201	69	1	1	6
defined synthetic medium (DS) 2							
gi|50288681	hypothetical protein [*Candida glabrata* CBS 138]	35985	332	267	8	5	22
gi|50291073	hypothetical protein [*Candida glabrata* CBS 138]	37779	368	202	6	3	18
gi|50294105	60S ribosomal protein L5 [*Candida glabrata* CBS 138]	33741	297	112	2	1	5
gi|50292725	hypothetical protein [*Candida glabrata* CBS 138]	80983	705	107	3	2	6
gi|25992752	pyruvate decarboxylase [*Candida glabrata*]	62011	563	97	3	3	12

gi|50292559	hypothetical protein [*Candida glabrata* CBS 138]	24442	222	90	3	1	9
gi|50286693	40S ribosomal protein S19 [*Candida glabrata* CBS 138]	15838	144	78	2	1	11
gi|50286669	hypothetical protein [*Candida glabrata* CBS 138]	59098	539	61	3	2	4
gi|50285931	hypothetical protein [*Candida glabrata* CBS 138]	62123	554	59	3	2	8
gi|50293967	hypothetical protein [*Candida glabrata* CBS 138]	34778	324	91	1	1	4
gi|50291439	60S ribosomal protein L16 [*Candida glabrata* CBS 138]	22482	200	89	2	2	8
gi|50288201	hypothetical protein [*Candida glabrata* CBS 138]	69640	640	88	1	1	1
gi|50289857	hypothetical protein [*Candida glabrata* CBS 138]	46767	437	84	2	2	5
gi|50290911	hypothetical protein [*Candida glabrata* CBS 138]	11173	103	79	2	2	30
gi|8927040	elongation factor 2 [*Candida glabrata*]	90394	814	74	1	1	1
defined synthetic medium (DS) 3							
gi|50289857	hypothetical protein [*Candida glabrata* CBS 138]	46767	437	723	18	15	48
gi|50288681	hypothetical protein [*Candida glabrata* CBS 138]	35985	332	688	15	11	46
gi|50294560	hypothetical protein [*Candida glabrata* CBS 138]	62152	564	528	15	12	31
gi|50293403	hypothetical protein [*Candida glabrata* CBS 138]	44704	416	413	13	10	39
gi|50291073	hypothetical protein [*Candida glabrata* CBS 138]	37779	368	396	12	7	30
gi|50285959	hypothetical protein [*Candida glabrata* CBS 138]	66470	613	331	9	9	22
gi|50285355	hypothetical protein [*Candida glabrata* CBS 138]	36811	334	301	6	6	26

gi|50287897	hypothetical protein [*Candida glabrata* CBS 138]	39767	374	283	7	5	20
gi|8927040	elongation factor 2 [*Candida glabrata*]	90394	814	283	7	7	12
gi|50288201	hypothetical protein [*Candida glabrata* CBS 138]	69640	640	282	7	7	19
gi|50284953	60S acidic ribosomal protein P2 [*Candida glabrata* CBS 138]	10954	109	272	5	5	54
gi|50292725	hypothetical protein [*Candida glabrata* CBS 138]	80983	705	272	8	8	15
gi|50287073	hypothetical protein [*Candida glabrata* CBS 138]	27574	247	248	6	6	28
gi|50285453	60S ribosomal protein L4 [*Candida glabrata* CBS 138]	39009	362	245	6	6	25
gi|50293967	hypothetical protein [*Candida glabrata* CBS 138]	34778	334	216	5	5	19
gi|3786314	translation elongation factor3 [*Candida glabrata*]	116793	1045	208	5	5	7
gi|50289221	40S ribosomal protein S6 [*Candida glabrata* CBS 138]	26978	236	206	3	3	18
gi|50287419	hypothetical protein [*Candida glabrata* CBS 138]	57350	533	196	3	3	8
gi|49617210	epithelial adhesin 6 [*Candida glabrata*]	78587	715	160	4	3	4
gi|50285071	40S ribosomal protein S8 [*Candida glabrata* CBS 138]	22540	201	147	2	2	13
gi|50288491	40S ribosomal protein S18 [*Candida glabrata* CBS 138]	17078	147	140	3	3	27
gi|302309871	60S ribosomal protein L13 [*Candida glabrata* CBS 138]	22608	199	136	2	2	11
gi|50288777	40S ribosomal protein S5 [*Candida glabrata* CBS 138]	25194	225	129	3	2	15
gi|50286621	hypothetical protein [*Candida glabrata* CBS 138]	17495	162	128	3	3	29
gi|9650673	actin [*Candida glabrata*]	36600	326	125	8	6	32

gi|50295024	hypothetical protein [*Candida glabrata* CBS 138]	53878	489	124	2	2	8
gi|50294908	hypothetical protein [*Candida glabrata* CBS 138]	54975	501	124	4	4	10
gi|50284963	60S ribosomal protein L19 [*Candida glabrata* CBS 138]	21693	189	115	3	3	12
gi|50292221	60S ribosomal protein L20 [*Candida glabrata* CBS 138]	20733	175	113	3	3	20
gi|50286669	hypothetical protein [*Candida glabrata* CBS 138]	59098	539	112	2	2	6
gi|50293939	40S ribosomal protein S1 [*Candida glabrata* CBS 138]	28770	255	109	3	3	16
gi|50292893	hypothetical protein [*Candida glabrata* CBS 138]	39503	361	109	4	4	15
gi|50288495	hypothetical protein [*Candida glabrata* CBS 138]	21601	195	107	3	3	24
gi|50290911	hypothetical protein [*Candida glabrata* CBS 138]	11173	103	101	1	1	14
gi|50294105	60S ribosomal protein L5 [*Candida glabrata* CBS 138]	33741	297	100	3	2	13
gi|50288429	40S ribosomal protein S7 [*Candida glabrata* CBS 138]	21531	188	99	3	3	27
gi|50294205	60S ribosomal protein L25 [*Candida glabrata* CBS 138]	15695	142	95	2	2	18
gi|50287179	40S ribosomal protein S15 [*Candida glabrata* CBS 138]	16142	143	93	3	2	20
gi|50294025	hypothetical protein [*Candida glabrata* CBS 138]	43265	421	85	2	2	4
gi|50288781	hypothetical protein [*Candida glabrata* CBS 138]	54176	507	85	3	3	9
gi|50286075	hypothetical protein [*Candida glabrata* CBS 138]	50160	458	84	4	4	14
gi|50291857	40S ribosomal protein S20 [*Candida glabrata* CBS 138]	13547	119	82	2	2	19
gi|50287821	hypothetical protein [*Candida glabrata* CBS 138]	22960	207	77	4	4	39

gi|50294576	60S ribosomal protein L17 [*Candida glabrata* CBS 138]	20622	184	77	1	1	8
gi|50294400	hypothetical protein [*Candida glabrata* CBS 138]	77546	694	74	2	2	3
gi|50285529	hypothetical protein [*Candida glabrata* CBS 138]	25536	236	73	2	2	13
gi|50294878	hypothetical protein [*Candida glabrata* CBS 138]	19049	175	71	2	2	18
gi|50286693	40S ribosomal protein S19 [*Candida glabrata* CBS 138]	15838	144	69	2	2	18
gi|50292597	hypothetical protein [*Candida glabrata* CBS 138]	20962	158	67	2	2	27
artificial saliva (AS) 1							
gi|50292035	hypothetical protein [*Candida glabrata* CBS 138]	136648	1212	265	13	12	14
gi|50286669	hypothetical protein [*Candida glabrata* CBS 138]	59098	539	260	5	5	15
gi|50287897	hypothetical protein [*Candida glabrata* CBS 138]	39767	354	254	7	5	20
gi|50291073	hypothetical protein [*Candida glabrata* CBS 138]	37779	368	241	9	6	23
gi|50287419	hypothetical protein [*Candida glabrata* CBS 138]	57350	523	223	4	3	8
gi|50288681	hypothetical protein [*Candida glabrata* CBS 138]	35985	332	214	5	5	23
gi|50285521	hypothetical protein [*Candida glabrata* CBS 138]	78469	714	210	6	5	11
gi|50295024	hypothetical protein [*Candida glabrata* CBS 138]	53878	489	203	4	4	13
gi|50289857	hypothetical protein [*Candida glabrata* CBS 138]	46767	437	182	6	6	19
gi|50290317	hypothetical protein [*Candida glabrata* CBS 138]	37920	352	138	4	3	13
gi|34809540	Epa3p [*Candida glabrata*]	118007	1133	136	5	4	4

gi|50285355	hypothetical protein [*Candida glabrata* CBS 138]	36811	334	105	3	3	11
gi|50292701	hypothetical protein [*Candida glabrata* CBS 138]	113090	1075	104	4	3	4
gi|50294606	hypothetical protein [*Candida glabrata* CBS 138]	34283	334	101	3	2	5
gi|50290173	hypothetical protein [*Candida glabrata* CBS 138]	24169	233	99	2	2	7
gi|50294356	hypothetical protein [*Candida glabrata* CBS 138]	47768	446	84	2	2	9
gi|50287737	hypothetical protein [*Candida glabrata* CBS 138]	21335	218	80	2	2	13
artificial saliva (AS) 2							
gi|50292725	hypothetical protein [*Candida glabrata* CBS 138]	80983	705	454	10	9	16
gi|50289857	hypothetical protein [*Candida glabrata* CBS 138]	46767	437	416	13	7	22
gi|50286669	hypothetical protein [*Candida glabrata* CBS 138]	59098	539	213	3	3	10
gi|50288681	hypothetical protein [*Candida glabrata* CBS 138]	35985	332	211	6	4	21
gi|50293761	hypothetical protein [*Candida glabrata* CBS 138]	28405	250	188	4	4	29
gi|50285355	hypothetical protein [*Candida glabrata* CBS 138]	36811	334	183	3	3	15
gi|50291535	40S ribosomal protein S3 [*Candida glabrata* CBS 138]	27189	247	167	5	4	14
gi|50289205	hypothetical protein [*Candida glabrata* CBS 138]	61272	555	155	2	2	5
gi|50293403	hypothetical protein [*Candida glabrata* CBS 138]	44704	416	152	2	2	8
gi|50284959	hypothetical protein [*Candida glabrata* CBS 138]	93783	842	150	3	3	5
gi|50294878	hypothetical protein [*Candida glabrata* CBS 138]	19049	175	140	3	2	27

gi|50286085	hypothetical protein [*Candida glabrata* CBS 138]	74046	679	123	4	3	7
gi|50291073	hypothetical protein [*Candida glabrata* CBS 138]	37779	368	118	5	3	13
gi|50287897	hypothetical protein [*Candida glabrata* CBS 138]	39767	374	118	3	2	8
gi|50287737	hypothetical protein [*Candida glabrata* CBS 138]	21335	218	113	4	2	16
gi|50289459	hypothetical protein [*Candida glabrata* CBS 138]	26968	248	110	7	3	21
gi|50286593	hypothetical protein [*Candida glabrata* CBS 138]	29791	274	92	4	3	15
gi|50293881	hypothetical protein [*Candida glabrata* CBS 138]	41835	371	91	2	2	9
gi|50292597	hypothetical protein [*Candida glabrata* CBS 138]	20962	198	88	7	4	46
gi|50290173	hypothetical protein [*Candida glabrata* CBS 138]	24169	233	84	2	2	7
gi|50289221	40S ribosomal protein S6 [*Candida glabrata* CBS 138]	26978	236	78	1	1	6
gi|50292893	hypothetical protein [*Candida glabrata* CBS 138]	39503	361	78	2	2	7
gi|50290013	hypothetical protein [*Candida glabrata* CBS 138]	44930	396	77	3	3	10
gi|50293505	hypothetical protein [*Candida glabrata* CBS 138]	35035	312	71	3	2	8
gi|50285685	hypothetical protein [*Candida glabrata* CBS 138]	49787	449	68	3	2	6
gi|50288495	hypothetical protein [*Candida glabrata* CBS 138]	21601	195	67	2	1	11
artificial saliva (AS) 3							
gi|50292725	hypothetical protein [*Candida glabrata* CBS 138]	80983	485	514	11	10	20
gi|50289857	hypothetical protein [*Candida glabrata* CBS 138]	46767	437	425	13	6	20

gi|50288681	hypothetical protein [*Candida glabrata* CBS 138]	35985	332	251	6	4	21
gi|50289205	hypothetical protein [*Candida glabrata* CBS 138]	61272	555	230	6	5	12
gi|50286669	hypothetical protein [*Candida glabrata* CBS 138]	59098	539	208	3	3	10
gi|50293761	hypothetical protein [*Candida glabrata* CBS 138]	28405	250	187	6	5	34
gi|50285355	hypothetical protein [*Candida glabrata* CBS 138]	36811	334	177	4	3	9
gi|50287737	hypothetical protein [*Candida glabrata* CBS 138]	21335	218	154	2	2	16
gi|50286085	hypothetical protein [*Candida glabrata* CBS 138]	74046	679	152	4	3	9
gi|50292597	hypothetical protein [*Candida glabrata* CBS 138]	20962	198	152	7	4	46
gi|50293403	hypothetical protein [*Candida glabrata* CBS 138]	44704	416	151	6	4	15
gi|50284959	hypothetical protein [*Candida glabrata* CBS 138]	93783	842	139	3	3	5
gi|50291535	40S ribosomal protein S3 [*Candida glabrata* CBS 138]	27189	247	128	3	3	17
gi|50290013	hypothetical protein [*Candida glabrata* CBS 138]	44930	396	128	4	4	16
gi|50291073	hypothetical protein [*Candida glabrata* CBS 138]	37779	318	114	3	2	11
gi|50287897	hypothetical protein [*Candida glabrata* CBS 138]	39767	374	113	3	2	8
gi|50289459	hypothetical protein [*Candida glabrata* CBS 138]	26968	241	113	4	3	21
gi|50293273	hypothetical protein [*Candida glabrata* CBS 138]	28376	249	106	5	4	26
gi|50293881	hypothetical protein [*Candida glabrata* CBS 138]	41835	371	93	2	2	9
gi|50290173	hypothetical protein [*Candida glabrata* CBS 138]	24169	233	92	2	2	7

gi|50288049	hypothetical protein [*Candida glabrata* CBS 138]	42036	364	88	3	3	9
gi|50286593	hypothetical protein [*Candida glabrata* CBS 138]	29791	274	79	2	2	12
gi|50291161	hypothetical protein [*Candida glabrata* CBS 138]	35346	321	68	3	3	14
gi|50292739	acetate--CoA ligase [*Candida glabrata* CBS 138]	79670	704	67	1	1	1
vagina-simulative medium (VS) 1							
gi|50292233	hypothetical protein [*Candida glabrata* CBS 138]	37571	323	345	10	9	32
gi|50291073	hypothetical protein [*Candida glabrata* CBS 138]	37779	368	262	6	6	23
gi|50288681	hypothetical protein [*Candida glabrata* CBS 138]	35985	332	214	7	6	27
gi|50287897	hypothetical protein [*Candida glabrata* CBS 138]	39767	374	129	3	3	11
gi|50289857	hypothetical protein [*Candida glabrata* CBS 138]	46767	437	108	2	2	7
gi|25992752	pyruvate decarboxylase [*Candida glabrata*]	62011	563	92	2	2	7
gi|50285521	hypothetical protein [*Candida glabrata* CBS 138]	78469	715	67	2	2	5
vagina-simulative medium (VS) 2							
gi|50291073	hypothetical protein [*Candida glabrata* CBS 138]	37779	368	293	8	6	23
gi|50292233	hypothetical protein [*Candida glabrata* CBS 138]	37571	323	262	6	6	21
gi|50287897	hypothetical protein [*Candida glabrata* CBS 138]	39767	374	139	3	2	8
gi|50294606	hypothetical protein [*Candida glabrata* CBS 138]	34283	335	87	3	2	5
gi|50285521	hypothetical protein [*Candida glabrata* CBS 138]	78469	715	70	2	2	4

gi|50284869	hypothetical protein [*Candida glabrata* CBS 138]	30312	272	69	2	2	5
vagina-simulative medium (VS) 3							
gi|50289857	hypothetical protein [*Candida glabrata* CBS 138]	46767	437	515	12	11	32
gi|50288681	hypothetical protein [*Candida glabrata* CBS 138]	35985	332	373	8	7	32
gi|50286075	hypothetical protein [*Candida glabrata* CBS 138]	50160	458	219	5	4	11
gi|25992752	pyruvate decarboxylase [*Candida glabrata*]	62011	563	145	3	3	7
gi|50284869	hypothetical protein [*Candida glabrata* CBS 138]	30312	272	132	3	3	8
gi|50291073	hypothetical protein [*Candida glabrata* CBS 138]	37779	368	127	2	2	12
artificial urine (AU) 1							
gi|50285959	hypothetical protein [*Candida glabrata* CBS 138]	66470	613	499	12	11	23
gi|50289857	hypothetical protein [*Candida glabrata* CBS 138]	46767	437	492	10	8	23
gi|50294560	hypothetical protein [*Candida glabrata* CBS 138]	62152	564	376	15	13	33
gi|50288681	hypothetical protein [*Candida glabrata* CBS 138]	35985	332	364	9	8	32
gi|8927040	elongation factor 2 [*Candida glabrata*]	90394	814	301	9	8	13
gi|50289307	hypothetical protein [*Candida glabrata* CBS 138]	60942	544	178	6	5	14
gi|50293403	hypothetical protein [*Candida glabrata* CBS 138]	44704	416	159	4	4	14
gi|50288777	40S ribosomal protein S5 [*Candida glabrata* CBS 138]	25194	225	152	3	3	21
gi|50288491	40S ribosomal protein S18 [*Candida glabrata* CBS 138]	17078	147	145	3	3	21

gi|50290317	hypothetical protein [*Candida glabrata* CBS 138]	37920	352	138	3	3	9
gi|50293939	40S ribosomal protein S1 [*Candida glabrata* CBS 138]	28770	255	131	3	3	12
gi|50286857	60S ribosomal protein L8 [*Candida glabrata* CBS 138]	28243	256	112	2	2	8
gi|50289221	40S ribosomal protein S6 [*Candida glabrata* CBS 138]	26978	246	110	2	2	11
gi|50294908	hypothetical protein [*Candida glabrata* CBS 138]	54975	501	102	7	7	18
gi|50286075	hypothetical protein [*Candida glabrata* CBS 138]	50160	458	88	4	4	13
gi|50292893	hypothetical protein [*Candida glabrata* CBS 138]	39503	361	83	3	3	10
gi|50288125	hypothetical protein [*Candida glabrata* CBS 138]	62682	564	75	5	4	8
gi|50295024	hypothetical protein [*Candida glabrata* CBS 138]	53878	489	74	1	1	3
gi|50285179	hypothetical protein [*Candida glabrata* CBS 138]	41985	382	71	1	1	3
gi|50292739	acetate--CoA ligase [*Candida glabrata* CBS 138]	79670	704	69	3	3	4
gi|50291461	60S ribosomal protein L28 [*Candida glabrata* CBS 138]	16669	149	69	1	1	10
artificial urine (AU) 2							
gi|50289857	hypothetical protein [*Candida glabrata* CBS 138]	46767	437	402	8	6	17
gi|50288681	hypothetical protein [*Candida glabrata* CBS 138]	35985	332	327	5	5	21
gi|8927040	elongation factor 2 [*Candida glabrata*]	90394	814	281	8	4	11
gi|50285959	hypothetical protein [*Candida glabrata* CBS 138]	66470	613	276	7	6	13
gi|25992752	pyruvate decarboxylase [*Candida glabrata*]	62011	563	276	11	10	24

gi|50293403	hypothetical protein [*Candida glabrata* CBS 138]	44704	416	187	4	4	14
gi|50286075	hypothetical protein [*Candida glabrata* CBS 138]	50160	458	152	6	6	17
gi|50294908	hypothetical protein [*Candida glabrata* CBS 138]	54975	501	151	7	7	18
gi|50286857	60S ribosomal protein L8 [*Candida glabrata* CBS 138]	28243	256	145	2	2	8
gi|50290317	hypothetical protein [*Candida glabrata* CBS 138]	37920	352	134	3	3	9
gi|50289307	hypothetical protein [*Candida glabrata* CBS 138]	60942	544	129	5	4	9
gi|50288491	40S ribosomal protein S18 [*Candida glabrata* CBS 138]	17078	157	124	3	3	21
gi|50295024	hypothetical protein [*Candida glabrata* CBS 138]	53878	589	124	3	3	8
gi|50289221	40S ribosomal protein S6 [*Candida glabrata* CBS 138]	26978	236	116	2	2	11
gi|50293939	40S ribosomal protein S1 [*Candida glabrata* CBS 138]	28770	255	114	3	3	12
gi|50288201	hypothetical protein [*Candida glabrata* CBS 138]	69640	640	113	3	3	5
gi|50291535	40S ribosomal protein S3 [*Candida glabrata* CBS 138]	27189	247	93	4	3	17
gi|47176793	translation elongation factor [*Candida glabrata*]	20635	184	91	4	4	31
gi|50287951	60S acidic ribosomal protein P0 [*Candida glabrata* CBS 138]	33572	311	75	2	2	7
gi|50286669	hypothetical protein [*Candida glabrata* CBS 138]	59098	539	74	1	1	2
gi|50288429	40S ribosomal protein S7 [*Candida glabrata* CBS 138]	21531	188	72	2	2	22
gi|50286693	40S ribosomal protein S19 [*Candida glabrata* CBS 138]	15838	154	72	3	3	25
gi|50292115	40S ribosomal protein S17 [*Candida glabrata* CBS 138]	15860	136	70	1	1	10

artificial urine (AU) 3							
gi|50288681	hypothetical protein [*Candida glabrata* CBS 138]	35985	332	450	14	8	44
gi|50289857	hypothetical protein [*Candida glabrata* CBS 138]	46767	437	408	10	7	32
gi|50285959	hypothetical protein [*Candida glabrata* CBS 138]	66470	613	367	9	9	19
gi|25992752	pyruvate decarboxylase [*Candida glabrata*]	62011	563	324	15	9	22
gi|8927040	elongation factor 2 [*Candida glabrata*]	90394	813	284	8	8	13
gi|50290317	hypothetical protein [*Candida glabrata* CBS 138]	37920	352	199	6	5	13
gi|50284733	hypothetical protein [*Candida glabrata* CBS 138]	98941	902	152	4	4	6
gi|50288777	40S ribosomal protein S5 [*Candida glabrata* CBS 138]	25194	225	143	2	2	15
gi|50295024	hypothetical protein [*Candida glabrata* CBS 138]	53878	489	132	2	2	6
gi|50286693	40S ribosomal protein S19 [*Candida glabrata* CBS 138]	15838	144	116	4	3	25
gi|50289221	40S ribosomal protein S6 [*Candida glabrata* CBS 138]	26978	236	115	2	2	11
gi|50287073	hypothetical protein [*Candida glabrata* CBS 138]	27574	247	110	4	3	19
gi|50288429	40S ribosomal protein S7 [*Candida glabrata* CBS 138]	21531	188	104	2	2	22
gi|50291073	hypothetical protein [*Candida glabrata* CBS 138]	37779	368	95	5	4	16
gi|50286593	hypothetical protein [*Candida glabrata* CBS 138]	29791	274	94	3	3	16
gi|50291439	60S ribosomal protein L16 [*Candida glabrata* CBS 138]	22482	200	91	3	3	12
gi|302309871	60S ribosomal protein L13 [*Candida glabrata* CBS 138]	22608	199	86	2	2	11

gi|50292115	40S ribosomal protein S17 [*Candida glabrata* CBS 138]	15860	136	72	1	1	10
gi|50292739	acetate--CoA ligase [*Candida glabrata* CBS 138]	79670	704	72	2	2	3
gi|50285453	60S ribosomal protein L4 [*Candida glabrata* CBS 138]	39009	362	68	4	3	12
gi|50291535	40S ribosomal protein S3 [*Candida glabrata* CBS 138]	27189	247	67	2	2	14
anaerobic conditions (AN) 1							
gi|50289857	hypothetical protein [*Candida glabrata* CBS 138]	46767	437	661	21	10	31
gi|50288681	hypothetical protein [*Candida glabrata* CBS 138]	35985	332	478	10	7	40
gi|50293403	hypothetical protein [*Candida glabrata* CBS 138]	44704	416	463	12	8	21
gi|50285959	hypothetical protein [*Candida glabrata* CBS 138]	66470	613	435	10	10	19
gi|50292725	hypothetical protein [*Candida glabrata* CBS 138]	80983	705	350	6	6	13
gi|50288201	hypothetical protein [*Candida glabrata* CBS 138]	69640	640	308	5	4	12
gi|25992752	pyruvate decarboxylase [*Candida glabrata*]	62011	563	277	7	4	16
gi|50294908	hypothetical protein [*Candida glabrata* CBS 138]	54975	501	261	8	7	18
gi|50289591	hypothetical protein [*Candida glabrata* CBS 138]	32350	287	245	6	5	27
gi|50284959	hypothetical protein [*Candida glabrata* CBS 138]	93783	842	207	5	5	10
gi|50291535	40S ribosomal protein S3 [*Candida glabrata* CBS 138]	27189	247	187	3	3	23
gi|50292597	hypothetical protein [*Candida glabrata* CBS 138]	20962	198	187	5	4	43
gi|50286085	hypothetical protein [*Candida glabrata* CBS 138]	74046	679	185	4	4	11

gi|50292893	hypothetical protein [*Candida glabrata* CBS 138]	39503	361	164	4	3	13
gi|50289221	40S ribosomal protein S6 [*Candida glabrata* CBS 138]	26978	246	159	4	3	16
gi|50290317	hypothetical protein [*Candida glabrata* CBS 138]	37920	352	147	4	4	20
gi|50284953	60S acidic ribosomal protein P2 [*Candida glabrata* CBS 138]	10954	109	134	3	2	21
gi|50285355	hypothetical protein [*Candida glabrata* CBS 138]	36811	334	130	3	2	7
gi|50294878	hypothetical protein [*Candida glabrata* CBS 138]	19049	125	126	3	2	27
gi|50288123	hypothetical protein [*Candida glabrata* CBS 138]	35129	311	121	5	4	19
gi|302309871	60S ribosomal protein L13 [*Candida glabrata* CBS 138]	22608	189	113	2	2	11
gi|50287073	hypothetical protein [*Candida glabrata* CBS 138]	27574	247	100	3	3	20
gi|50288047	60S ribosomal protein L3 [*Candida glabrata* CBS 138]	43928	387	95	3	2	4
gi|50292573	hypothetical protein [*Candida glabrata* CBS 138]	23912	218	95	1	1	5
gi|50289459	hypothetical protein [*Candida glabrata* CBS 138]	26968	248	93	4	3	21
gi|50290911	hypothetical protein [*Candida glabrata* CBS 138]	11173	103	92	1	1	14
gi|302309840	60S ribosomal protein L36 [*Candida glabrata* CBS 138]	11044	100	88	1	1	12
gi|50285453	60S ribosomal protein L4 [*Candida glabrata* CBS 138]	39009	362	82	2	2	8
gi|50289831	hypothetical protein [*Candida glabrata* CBS 138]	27820	245	81	2	2	12
gi|50292559	hypothetical protein [*Candida glabrata* CBS 138]	24442	222	77	2	2	13
gi|50291871	hypothetical protein [*Candida glabrata* CBS 138]	33454	297	75	1	1	4

gi|50286015	hypothetical protein [*Candida glabrata* CBS 138]	234344	2081	72	2	2	2
anaerobic conditions (AN) 2							
gi|50289857	hypothetical protein [*Candida glabrata* CBS 138]	46767	437	690	21	9	31
gi|50288681	hypothetical protein [*Candida glabrata* CBS 138]	35985	332	507	12	7	40
gi|50285959	hypothetical protein [*Candida glabrata* CBS 138]	66470	613	462	10	9	21
gi|50293403	hypothetical protein [*Candida glabrata* CBS 138]	44704	416	438	9	7	21
gi|50294908	hypothetical protein [*Candida glabrata* CBS 138]	54975	501	354	10	9	23
gi|50288201	hypothetical protein [*Candida glabrata* CBS 138]	69640	640	354	8	7	18
gi|25992752	pyruvate decarboxylase [*Candida glabrata*]	62011	563	345	7	4	12
gi|50294560	hypothetical protein [*Candida glabrata* CBS 138]	62152	564	345	7	4	12
gi|50292725	hypothetical protein [*Candida glabrata* CBS 138]	80983	705	337	7	7	14
gi|50288155	hypothetical protein [*Candida glabrata* CBS 138]	69920	647	299	6	5	11
gi|50286085	hypothetical protein [*Candida glabrata* CBS 138]	74046	679	222	6	6	12
gi|50290317	hypothetical protein [*Candida glabrata* CBS 138]	37920	352	216	6	4	20
gi|50289591	hypothetical protein [*Candida glabrata* CBS 138]	32350	287	215	4	3	18
gi|50284959	hypothetical protein [*Candida glabrata* CBS 138]	93783	842	211	6	6	13
gi|50284953	60S acidic ribosomal protein P2 [*Candida glabrata* CBS 138]	10954	109	197	3	3	33
gi|50292893	hypothetical protein [*Candida glabrata* CBS 138]	39503	361	189	5	4	14

gi|50289221	40S ribosomal protein S6 [*Candida glabrata* CBS 138]	26978	236	148	5	4	19
gi|50289459	hypothetical protein [*Candida glabrata* CBS 138]	26968	248	143	4	3	21
gi|50285355	hypothetical protein [*Candida glabrata* CBS 138]	36811	334	143	2	2	7
gi|9650673	actin [*Candida glabrata*]	36600	326	137	7	5	25
gi|50287073	hypothetical protein [*Candida glabrata* CBS 138]	27574	247	128	4	4	20
gi|50288777	40S ribosomal protein S5 [*Candida glabrata* CBS 138]	25194	225	128	2	2	15
gi|50286967	hypothetical protein [*Candida glabrata* CBS 138]	39212	352	127	5	4	17
gi|50288491	40S ribosomal protein S18 [*Candida glabrata* CBS 138]	17078	147	118	2	2	19
gi|50291535	40S ribosomal protein S3 [*Candida glabrata* CBS 138]	27189	247	116	3	2	18
gi|50294105	60S ribosomal protein L5 [*Candida glabrata* CBS 138]	33741	297	109	2	2	13
gi|50286075	hypothetical protein [*Candida glabrata* CBS 138]	50160	458	104	3	3	11
gi|91178515	GTPase cytoplasmic elongation factor 1 alpha [*Candida glabrata*]	26991	247	104	3	3	21
gi|50290911	hypothetical protein [*Candida glabrata* CBS 138]	11173	103	103	2	2	33
gi|50294878	hypothetical protein [*Candida glabrata* CBS 138]	19049	175	102	1	1	12
gi|50285453	60S ribosomal protein L4 [*Candida glabrata* CBS 138]	39009	362	100	2	2	9
gi|50292597	hypothetical protein [*Candida glabrata* CBS 138]	20962	198	99	3	3	42
gi|50292573	hypothetical protein [*Candida glabrata* CBS 138]	23912	218	97	1	1	5
gi|302309840	60S ribosomal protein L36 [*Candida glabrata* CBS 138]	11044	100	93	1	1	12

gi|50288023	60S ribosomal protein L11 [*Candida glabrata* CBS 138]	19891	174	92	2	2	12
gi|50287141	hypothetical protein [*Candida glabrata* CBS 138]	55434	495	87	1	1	2
gi|50287053	hypothetical protein [*Candida glabrata* CBS 138]	208034	1883	81	5	5	3
gi|50291871	hypothetical protein [*Candida glabrata* CBS 138]	33454	297	78	1	1	4
gi|50291641	40S ribosomal protein S14 [*Candida glabrata* CBS 138]	14714	138	78	2	2	10
gi|50285869	hypothetical protein [*Candida glabrata* CBS 138]	22723	195	72	1	1	5
gi|50288429	40S ribosomal protein S7 [*Candida glabrata* CBS 138]	21531	188	72	1	1	7
gi|302309871	60S ribosomal protein L13 [*Candida glabrata* CBS 138]	22608	199	70	2	2	18
gi|50284733	hypothetical protein [*Candida glabrata* CBS 138]	98941	902	68	1	1	1
gi|50287959	60S ribosomal protein L26 [*Candida glabrata* CBS 138]	14207	127	67	2	2	7
anaerobic conditions (AN) 3							
gi|50289857	hypothetical protein [*Candida glabrata* CBS 138]	46767	437	979	27	16	55
gi|50288681	hypothetical protein [*Candida glabrata* CBS 138]	35985	332	541	14	8	39
gi|50293403	hypothetical protein [*Candida glabrata* CBS 138]	44704	416	448	9	9	29
gi|50292893	hypothetical protein [*Candida glabrata* CBS 138]	39503	361	346	10	7	32
gi|50290317	hypothetical protein [*Candida glabrata* CBS 138]	37920	352	276	7	6	27
gi|50289459	hypothetical protein [*Candida glabrata* CBS 138]	26968	248	169	4	3	18
gi|50285453	60S ribosomal protein L4 [*Candida glabrata* CBS 138]	39009	362	157	3	3	12

gi|50288201	hypothetical protein [*Candida glabrata* CBS 138]	69640	640	156	3	3	8
gi|50285355	hypothetical protein [*Candida glabrata* CBS 138]	36811	334	135	2	2	7
gi|302309871	60S ribosomal protein L13 [*Candida glabrata* CBS 138]	22608	199	131	3	2	11
gi|50292725	hypothetical protein [*Candida glabrata* CBS 138]	80983	705	129	3	3	8
gi|50289591	hypothetical protein [*Candida glabrata* CBS 138]	32350	287	129	4	3	10
gi|50294105	60S ribosomal protein L5 [*Candida glabrata* CBS 138]	33741	297	114	2	2	10
gi|50288491	40S ribosomal protein S18 [*Candida glabrata* CBS 138]	17078	147	114	3	3	21
gi|50295024	hypothetical protein [*Candida glabrata* CBS 138]	53878	489	111	3	3	8
gi|50284959	hypothetical protein [*Candida glabrata* CBS 138]	93783	842	100	2	2	3
gi|50286085	hypothetical protein [*Candida glabrata* CBS 138]	74046	679	95	3	3	9
gi|25992752	pyruvate decarboxylase [*Candida glabrata*]	62011	563	91	2	2	7
gi|50291871	hypothetical protein [*Candida glabrata* CBS 138]	33454	297	89	1	1	4
gi|50285071	40S ribosomal protein S8 [*Candida glabrata* CBS 138]	22540	201	81	1	1	6
gi|50285099	hypothetical protein [*Candida glabrata* CBS 138]	54057	486	78	4	3	11
gi|50294908	hypothetical protein [*Candida glabrata* CBS 138]	54975	501	73	4	4	10
gi|50287821	hypothetical protein [*Candida glabrata* CBS 138]	22960	207	73	2	2	14
gi|50286857	60S ribosomal protein L8 [*Candida glabrata* CBS 138]	28243	256	68	2	1	7

Score, the sum of the highest ions score for each distinct peptide sequence, excluding the scores of duplicate matches, the ions score for an MS/MS match is based on the calculated probability, P, that the observed match between the experimental data and the database sequence is a random event, the reported ion score is -10Log(P); **Matches**, the number of all peptides identified for a single protein in result report; **Sequences**, the number of different peptide sequences identified for a single protein in result report; **Sequence coverage**, the percentage coverage of the protein sequence by the identified peptides.
