# Supplementary material for: Moonlighting proteins are variably exposed at the cell surfaces of Candida glabrata, Candida parapsilosis and Candida tropicalis under certain growth conditions
Source: BMC Microbiol. 2019 Jul 3;19:149. doi: 10.1186/s12866-019-1524-5 (PMC6609379; doi:10.1186/s12866-019-1524-5)
Supplement: Supplementary file 3 — Table S3. Mass spectrometry identification of C. parapsilosis proteins present at the cell surface under different growth conditions. (PDF 261 kb) [file 12866_2019_1524_MOESM3_ESM.pdf]

Supplementary table 3. Mass spectrometry identification of *C. parapsilosis* proteins present at the cell surface under different growth conditions.

Cell surface shaving of fungal cells with trypsin and the additional digestion of the obtained proteins for 24 hours was performed. The resulting peptides were analyzed using the Dionex Ultimate 3000 UHPLC system coupled to an HCTUltra ETDII mass spectrometer. The obtained lists of peaks were searched against the NCBI protein database using an in-house Mascot server.

**Accession number**	**Protein**	**Molecular mass [Da]**	**Number of amino acids**	**Score**	**Matches**	**Sequences**	**Sequence coverage [%]**
defined synthetic medium (DS) 1							
gi|354544127	hypothetical protein CPAR2_108890 [*Candida parapsilosis*]	118910	1083	529	14	12	14
gi|354547997	hypothetical protein CPAR2_405360 [*Candida parapsilosis*]	38031	354	442	8	7	29
gi|354545888	hypothetical protein CPAR2_202600 [*Candida parapsilosis*]	35921	324	419	6	6	23
gi|354546348	hypothetical protein CPAR2_207210 [*Candida parapsilosis*]	46995	439	393	9	9	29
gi|354548203	hypothetical protein CPAR2_407410 [*Candida parapsilosis*]	39522	372	362	7	5	25
gi|354544096	hypothetical protein CPAR2_108560 [*Candida parapsilosis*]	44734	433	297	8	6	17
gi|354545372	hypothetical protein CPAR2_806490 [*Candida parapsilosis*]	41477	400	288	5	5	13
gi|354543937	hypothetical protein CPAR2_106940 [*Candida parapsilosis*]	69920	646	272	5	5	12
gi|354547255	hypothetical protein CPAR2_502140 [*Candida parapsilosis*]	61228	584	268	8	5	10
gi|354547623	hypothetical protein CPAR2_401600 [*Candida parapsilosis*]	34120	308	255	6	6	26
gi|354544206	hypothetical protein CPAR2_109660 [*Candida parapsilosis*]	60797	566	230	3	3	7

gi|354543842	hypothetical protein CPAR2_106000 [*Candida parapsilosis*]	49102	424	229	6	6	19
gi|354545590	hypothetical protein CPAR2_808670 [*Candida parapsilosis*]	36264	336	227	7	7	29
gi|354547257	hypothetical protein CPAR2_502160 [*Candida parapsilosis*]	49909	463	143	4	4	13
gi|354548325	hypothetical protein CPAR2_700650 [*Candida parapsilosis*]	34248	328	143	3	2	9
gi|354547143	hypothetical protein CPAR2_501020 [*Candida parapsilosis*]	62596	566	141	3	3	9
gi|354545390	hypothetical protein CPAR2_806670 [*Candida parapsilosis*]	53439	522	139	4	2	5
gi|3549613	ADP/ATP carrier protein [*Candida parapsilosis*]	32914	303	137	3	2	10
gi|354548565	hypothetical protein CPAR2_703150 [*Candida parapsilosis*]	81506	710	132	3	3	5
gi|354547686	hypothetical protein CPAR2_402220 [*Candida parapsilosis*]	53445	501	132	3	3	8
gi|354543976	hypothetical protein CPAR2_107330 [*Candida parapsilosis*]	37592	352	98	4	3	13
gi|354544910	hypothetical protein CPAR2_801850 [*Candida parapsilosis*]	41980	415	87	1	1	3
gi|354547945	hypothetical protein CPAR2_404840 [*Candida parapsilosis*]	21958	196	86	3	2	12
gi|354547549	hypothetical protein CPAR2_400860 [*Candida parapsilosis*]	52009	490	70	1	1	2
gi|354543158	hypothetical protein CPAR2_602950 [*Candida parapsilosis*]	45057	417	67	2	2	7
defined synthetic medium (DS) 2							
gi|354547997	hypothetical protein CPAR2_405360 [*Candida parapsilosis*]	38031	354	507	10	9	37
gi|354544127	hypothetical protein CPAR2_108890 [*Candida parapsilosis*]	118910	1093	487	15	13	16
gi|354546348	hypothetical protein CPAR2_207210 [*Candida parapsilosis*]	46995	439	408	8	8	33

gi|354548203	hypothetical protein CPAR2_407410 [*Candida parapsilosis*]	39522	372	390	7	5	19
gi|354544096	hypothetical protein CPAR2_108560 [*Candida parapsilosis*]	44734	433	366	9	7	18
gi|354545590	hypothetical protein CPAR2_808670 [*Candida parapsilosis*]	36264	336	289	8	8	32
gi|354545372	hypothetical protein CPAR2_806490 [*Candida parapsilosis*]	41477	400	286	7	5	13
gi|354545888	hypothetical protein CPAR2_202600 [*Candida parapsilosis*]	35921	324	284	5	5	20
gi|354547255	hypothetical protein CPAR2_502140 [*Candida parapsilosis*]	61228	584	265	6	4	10
gi|354543842	hypothetical protein CPAR2_106000 [*Candida parapsilosis*]	49102	424	244	6	6	16
gi|354543937	hypothetical protein CPAR2_106940 [*Candida parapsilosis*]	69920	646	239	6	6	14
gi|354544206	hypothetical protein CPAR2_109660 [*Candida parapsilosis*]	60797	566	159	2	2	4
gi|354547623	hypothetical protein CPAR2_401600 [*Candida parapsilosis*]	34120	308	159	3	3	7
gi|354547549	hypothetical protein CPAR2_400860 [*Candida parapsilosis*]	52009	490	154	2	2	5
gi|354545390	hypothetical protein CPAR2_806670 [*Candida parapsilosis*]	53439	522	148	3	2	5
gi|354548325	hypothetical protein CPAR2_700650 [*Candida parapsilosis*]	34248	328	140	3	2	9
gi|354547257	hypothetical protein CPAR2_502160 [*Candida parapsilosis*]	49909	463	125	5	4	13
gi|354547143	hypothetical protein CPAR2_501020 [*Candida parapsilosis*]	62596	566	119	3	3	7
gi|354544910	hypothetical protein CPAR2_801850 [*Candida parapsilosis*]	41980	415	94	1	1	3
gi|354543224	hypothetical protein CPAR2_603600 [*Candida parapsilosis*]	72175	627	68	3	3	8
defined synthetic medium (DS) 3							

gi|354546348	hypothetical protein CPAR2_207210 [*Candida parapsilosis*]	46995	439	541	13	12	42
gi|354545888	hypothetical protein CPAR2_202600 [*Candida parapsilosis*]	35921	324	483	9	9	35
gi|354547997	hypothetical protein CPAR2_405360 [*Candida parapsilosis*]	38031	354	477	8	8	35
gi|354543937	hypothetical protein CPAR2_106940 [*Candida parapsilosis*]	69920	646	453	12	11	28
gi|354547686	hypothetical protein CPAR2_402220 [*Candida parapsilosis*]	53445	501	369	7	6	16
gi|354545372	hypothetical protein CPAR2_806490 [*Candida parapsilosis*]	41477	400	367	7	6	13
gi|354548565	hypothetical protein CPAR2_703150 [*Candida parapsilosis*]	81506	710	303	10	10	21
gi|354546195	hypothetical protein CPAR2_205670 [*Candida parapsilosis*]	66661	613	292	9	9	23
gi|354548203	hypothetical protein CPAR2_407410 [*Candida parapsilosis*]	39522	372	284	6	4	20
gi|3549613	ADP/ATP carrier protein [*Candida parapsilosis*]	32914	303	268	4	3	14
gi|354544206	hypothetical protein CPAR2_109660 [*Candida parapsilosis*]	60797	566	265	4	4	9
gi|354547336	hypothetical protein CPAR2_502950 [*Candida parapsilosis*]	98544	898	245	6	6	10
gi|354545590	hypothetical protein CPAR2_808670 [*Candida parapsilosis*]	36264	336	231	6	6	27
gi|354544910	hypothetical protein CPAR2_801850 [*Candida parapsilosis*]	41980	414	217	3	3	9
gi|354546787	hypothetical protein CPAR2_211630 [*Candida parapsilosis*]	93808	842	195	6	6	11
gi|354547414	hypothetical protein CPAR2_503730 [*Candida parapsilosis*]	21149	186	182	3	3	20
gi|354543842	hypothetical protein CPAR2_106000 [*Candida parapsilosis*]	49102	424	170	4	4	10
gi|354547255	hypothetical protein CPAR2_502140 [*Candida parapsilosis*]	61228	584	160	3	3	8

gi|354544127	hypothetical protein CPAR2_108890 [*Candida parapsilosis*]	118910	1093	143	6	4	4
gi|354546333	hypothetical protein CPAR2_207060 [*Candida parapsilosis*]	50479	458	138	4	4	10
gi|354547549	hypothetical protein CPAR2_400860 [*Candida parapsilosis*]	52009	490	138	3	3	8
gi|354544926	hypothetical protein CPAR2_802010 [*Candida parapsilosis*]	52049	466	131	4	3	10
gi|354543839	hypothetical protein CPAR2_105970 [*Candida parapsilosis*]	23238	202	125	2	2	10
gi|354545198	hypothetical protein CPAR2_804740 [*Candida parapsilosis*]	74602	681	118	4	4	11
gi|354545932	hypothetical protein CPAR2_203040 [*Candida parapsilosis*]	60457	562	115	3	3	8
gi|354544096	hypothetical protein CPAR2_108560 [*Candida parapsilosis*]	44734	433	110	3	2	8
gi|354548495	hypothetical protein CPAR2_702440 [*Candida parapsilosis*]	10951	111	90	1	1	14
gi|354544776	hypothetical protein CPAR2_800530 [*Candida parapsilosis*]	61945	555	86	3	3	9
gi|354543514	hypothetical protein CPAR2_102710 [*Candida parapsilosis*]	19981	183	83	1	1	7
gi|354544503	hypothetical protein CPAR2_302160 [*Candida parapsilosis*]	27165	236	80	2	2	13
gi|354545694	hypothetical protein CPAR2_200650 [*Candida parapsilosis*]	58133	520	80	1	1	2
gi|354545039	hypothetical protein CPAR2_803150 [*Candida parapsilosis*]	32818	299	78	1	1	6
gi|354543255	hypothetical protein CPAR2_100110 [*Candida parapsilosis*]	50537	464	74	1	1	3
gi|354543680	hypothetical protein CPAR2_104370 [*Candida parapsilosis*]	19880	174	72	2	2	13
gi|354545343	hypothetical protein CPAR2_806200 [*Candida parapsilosis*]	32161	285	70	1	1	3
gi|354548326	hypothetical protein CPAR2_700660 [*Candida parapsilosis*]	39330	353	69	3	3	14
artificial saliva (AS) 1							

gi|354546845	hypothetical protein CPAR2_212210 [*Candida parapsilosis*]	54072	497	373	12	12	36
gi|354548203	hypothetical protein CPAR2_407410 [*Candida parapsilosis*]	39522	372	215	6	4	20
gi|354547255	hypothetical protein CPAR2_502140 [*Candida parapsilosis*	61228	584	211	6	4	10
gi|354547299	hypothetical protein CPAR2_502580 [*Candida parapsilosis*]	43513	403	188	5	5	14
gi|354543937	hypothetical protein CPAR2_106940 [*Candida parapsilosis*]	69920	646	161	5	5	8
gi|354543976	hypothetical protein CPAR2_107330 [*Candida parapsilosis*]	37592	352	143	5	3	15
gi|354547091	hypothetical protein CPAR2_500500 [*Candida parapsilosis*]	36553	341	115	2	2	6
gi|354545372	hypothetical protein CPAR2_806490 [*Candida parapsilosis*]	41477	350	105	4	3	7
gi|354545390	hypothetical protein CPAR2_806670 [*Candida parapsilosis*]	53439	522	100	2	2	5
gi|354544776	hypothetical protein CPAR2_800530 [*Candida parapsilosis*]	61945	555	76	1	1	2
artificial saliva (AS) 2							
gi|354546845	hypothetical protein CPAR2_212210 [*Candida parapsilosis*]	54072	497	312	10	10	30
gi|354547255	hypothetical protein CPAR2_502140 [*Candida parapsilosis*]	61228	584	225	7	4	10
gi|354547299	hypothetical protein CPAR2_502580 [*Candida parapsilosis*]	43513	403	202	5	5	13
gi|354543976	hypothetical protein CPAR2_107330 [*Candida parapsilosis*]	37592	352	171	6	3	15
gi|354548203	hypothetical protein CPAR2_407410 [*Candida parapsilosis*]	39522	372	134	2	1	6
gi|354545390	hypothetical protein CPAR2_806670 [*Candida parapsilosis*]	53439	522	102	3	2	5
gi|354544206	hypothetical protein CPAR2_109660 [*Candida parapsilosis*]	60797	566	99	2	2	4
gi|354547091	hypothetical protein CPAR2_500500 [*Candida parapsilosis*]	36553	341	76	1	1	4
gi|354543937	hypothetical protein CPAR2_106940 [*Candida parapsilosis*]	69920	646	67	2	2	3
artificial saliva (AS) 3							
gi|354548203	hypothetical protein CPAR2_407410 [*Candida parapsilosis*]	39522	372	370	7	5	20
gi|354543976	hypothetical protein CPAR2_107330 [*Candida parapsilosis*]	37592	352	341	10	5	23
gi|354544096	hypothetical protein CPAR2_108560 [*Candida parapsilosis*]	44734	433	282	7	6	17
gi|354547255	hypothetical protein CPAR2_502140 [*Candida parapsilosis*]	61228	584	278	7	4	10
gi|354547091	hypothetical protein CPAR2_500500 [*Candida parapsilosis*]	36553	341	192	4	3	14
gi|354545372	hypothetical protein CPAR2_806490 [*Candida parapsilosis*]	41477	350	168	5	4	12
gi|354543842	hypothetical protein CPAR2_106000 [*Candida parapsilosis*]	49102	424	158	3	3	8
gi|354544206	hypothetical protein CPAR2_109660 [*Candida parapsilosis*]	60797	566	143	3	3	7

gi|354547623	hypothetical protein CPAR2_401600 [*Candida parapsilosis*]	34120	308	143	4	3	11
gi|354547549	hypothetical protein CPAR2_400860 [*Candida parapsilosis*]	52009	490	130	3	3	8
gi|354545390	hypothetical protein CPAR2_806670 [*Candida parapsilosis*]	53439	522	124	3	2	5
gi|354544910	hypothetical protein CPAR2_801850 [*Candida parapsilosis*]	41980	415	120	2	1	3
gi|354545615	hypothetical protein CPAR2_808920 [*Candida parapsilosis*]	94691	884	68	2	2	2
vagina-simulative medium (VS) 1							
gi|354548203	hypothetical protein CPAR2_407410 [*Candida parapsilosis*]	39522	372	369	6	5	19
gi|354546478	hypothetical protein CPAR2_208530 [*Candida parapsilosis*]	35667	326	114	2	2	6
gi|354545372	hypothetical protein CPAR2_806490 [*Candida parapsilosis*]	41477	400	99	2	2	7
gi|354543976	hypothetical protein CPAR2_107330 [*Candida parapsilosis*]	37592	352	84	2	2	9
gi|354547255	hypothetical protein CPAR2_502140 [*Candida parapsilosis*]	61228	584	80	1	1	2
vagina-simulative medium (VS) 2							
gi|354548203	hypothetical protein CPAR2_407410 [*Candida parapsilosis*]	39522	372	378	6	4	17
gi|354546478	hypothetical protein CPAR2_208530 [*Candida parapsilosis*]	35667	336	197	3	3	11
gi|354547255	hypothetical protein CPAR2_502140 [*Candida parapsilosis*	61228	584	142	2	2	6
gi|354545372	hypothetical protein CPAR2_806490 [*Candida parapsilosis*]	41477	400	101	2	2	7
gi|354545680	hypothetical protein CPAR2_200500 [*Candida parapsilosis*]	37725	325	76	1	1	4
gi|354545390	hypothetical protein CPAR2_806670 [*Candida parapsilosis*]	53439	513	73	3	2	5
gi|354543976	hypothetical protein CPAR2_107330 [*Candida parapsilosis*]	37592	352	70	3	2	9
vagina-simulative medium (VS) 3							
gi|354548203	hypothetical protein CPAR2_407410 [*Candida parapsilosis*]	39522	372	322	8	7	32
gi|354544910	hypothetical protein CPAR2_801850 [*Candida parapsilosis*]	41980	415	275	5	4	12
gi|354547255	hypothetical protein CPAR2_502140 [*Candida parapsilosis*]	61228	584	247	4	4	10
gi|354545372	hypothetical protein CPAR2_806490 [*Candida parapsilosis*]	41477	400	202	7	4	10
gi|354543976	hypothetical protein CPAR2_107330 [*Candida parapsilosis*]	37592	352	185	5	3	15
gi|354546348	hypothetical protein CPAR2_207210 [*Candida parapsilosis*]	46995	439	151	6	5	23
gi|354543937	hypothetical protein CPAR2_106940 [*Candida parapsilosis*]	69920	646	145	4	4	10
gi|354546478	hypothetical protein CPAR2_208530 [*Candida parapsilosis*]	35667	326	117	4	3	11
gi|354544206	hypothetical protein CPAR2_109660 [*Candida parapsilosis*]	60797	566	115	2	2	4

gi|354548565	hypothetical protein CPAR2_703150 [*Candida parapsilosis*]	81506	710	112	4	3	6
gi|354545590	hypothetical protein CPAR2_808670 [*Candida parapsilosis*]	36264	336	104	3	3	9
gi|354546284	hypothetical protein CPAR2_206570 [*Candida parapsilosis*]	6746	64	91	2	2	53
gi|354544096	hypothetical protein CPAR2_108560 [*Candida parapsilosis*]	44734	433	80	3	3	10
gi|354547091	hypothetical protein CPAR2_500500 [*Candida parapsilosis*]	36553	341	77	2	2	12
artificial urine (AU) 1							
gi|354543937	hypothetical protein CPAR2_106940 [*Candida parapsilosis*]	69920	646	145	3	2	4
gi|354543158	hypothetical protein CPAR2_602950 [*Candida parapsilosis*]	45057	417	137	4	4	11
gi|354545888	hypothetical protein CPAR2_202600 [*Candida parapsilosis*]	35921	324	132	3	3	12
gi|354546348	hypothetical protein CPAR2_207210 [*Candida parapsilosis*]	46995	439	120	2	2	6
gi|354545590	hypothetical protein CPAR2_808670 [*Candida parapsilosis*]	36264	336	118	3	2	10
gi|354547299	hypothetical protein CPAR2_502580 [*Candida parapsilosis*]	43513	403	108	3	3	7
gi|354545390	hypothetical protein CPAR2_806670 [*Candida parapsilosis*]	53439	522	95	2	2	5
gi|354544503	hypothetical protein CPAR2_302160 [*Candida parapsilosis*]	27165	236	95	2	2	8
gi|354543410	hypothetical protein CPAR2_101670 [*Candida parapsilosis*]	13287	127	79	2	2	17
artificial urine (AU) 2							
gi|354543937	hypothetical protein CPAR2_106940 [*Candida parapsilosis*]	69920	646	168	4	3	6
gi|354546348	hypothetical protein CPAR2_207210 [*Candida parapsilosis*]	46995	439	157	5	4	11
gi|354543158	hypothetical protein CPAR2_602950 [*Candida parapsilosis*]	45057	417	145	5	5	13
gi|354544503	hypothetical protein CPAR2_302160 [*Candida parapsilosis*]	27165	236	133	3	3	15
gi|354547299	hypothetical protein CPAR2_502580 [*Candida parapsilosis*]	43513	403	121	4	4	10
gi|354545590	hypothetical protein CPAR2_808670 [*Candida parapsilosis*]	36264	336	102	3	2	10
gi|354545390	hypothetical protein CPAR2_806670 [*Candida parapsilosis*]	53439	522	91	2	2	5
gi|354545888	hypothetical protein CPAR2_202600 [*Candida parapsilosis*]	35921	324	83	3	3	12
gi|354547143	hypothetical protein CPAR2_501020 [*Candida parapsilosis*]	62596	566	76	2	2	3
gi|354547586	hypothetical protein CPAR2_401230 [*Candida parapsilosis*]	39816	359	72	3	3	7
gi|354545451	hypothetical protein CPAR2_807280 [*Candida parapsilosis*]	27980	258	71	2	2	8
gi|354548524	hypothetical protein CPAR2_702740 [*Candida parapsilosis*]	17538	162	69	3	3	28
artificial urine (AU) 3							

gi|354546348	hypothetical protein CPAR2_207210 [*Candida parapsilosis*]	46995	439	411	11	10	28
gi|354547336	hypothetical protein CPAR2_502950 [*Candida parapsilosis*]	98544	898	387	8	8	10
gi|354543158	hypothetical protein CPAR2_602950 [*Candida parapsilosis*]	45057	417	348	12	9	31
gi|354543937	hypothetical protein CPAR2_106940 [*Candida parapsilosis*]	69920	646	299	4	4	8
gi|354546195	hypothetical protein CPAR2_205670 [*Candida parapsilosis*]	66661	613	245	6	6	14
gi|354547299	hypothetical protein CPAR2_502580 [*Candida parapsilosis*]	43513	403	197	5	5	13
gi|354547143	hypothetical protein CPAR2_501020 [*Candida parapsilosis*]	62596	566	171	4	3	7
gi|354547950	hypothetical protein CPAR2_404890 [*Candida parapsilosis*]	17744	165	136	6	3	32
gi|354545888	hypothetical protein CPAR2_202600 [*Candida parapsilosis*]	35921	324	110	3	2	7
gi|354546564	hypothetical protein CPAR2_209410 [*Candida parapsilosis*]	13048	112	106	2	2	22
gi|354545590	hypothetical protein CPAR2_808670 [*Candida parapsilosis*]	36264	336	100	10	4	17
gi|354544503	hypothetical protein CPAR2_302160 [*Candida parapsilosis*]	27165	236	94	2	2	8
gi|354544206	hypothetical protein CPAR2_109660 [*Candida parapsilosis*]	60797	566	81	1	1	2
gi|354547414	hypothetical protein CPAR2_503730 [*Candida parapsilosis*]	21149	186	73	1	1	6
anaerobic conditions (AN) 1							
i|354543937	hypothetical protein CPAR2_106940 [*Candida parapsilosis*]	69920	646	835	19	14	28
gi|354543158	hypothetical protein CPAR2_602950 [*Candida parapsilosis*]	45057	417	507	16	12	38
gi|354546348	hypothetical protein CPAR2_207210 [*Candida parapsilosis*]	46995	439	396	11	8	21
gi|354548565	hypothetical protein CPAR2_703150 [*Candida parapsilosis*]	81506	710	394	9	8	19
gi|354547143	hypothetical protein CPAR2_501020 [*Candida parapsilosis*]	62596	556	336	6	5	16
gi|354547586	hypothetical protein CPAR2_401230 [*Candida parapsilosis*]	39816	359	281	7	6	21
gi|354547299	hypothetical protein CPAR2_502580 [*Candida parapsilosis*]	43513	403	226	4	4	13
gi|354545888	hypothetical protein CPAR2_202600 [*Candida parapsilosis*]	35921	324	204	3	3	11
gi|354545590	hypothetical protein CPAR2_808670 [*Candida parapsilosis*]	36264	336	194	6	6	19
gi|354545521	hypothetical protein CPAR2_807980 [*Candida parapsilosis*]	26955	248	163	5	4	27
gi|354546805	hypothetical protein CPAR2_211810 [*Candida parapsilosis*]	27649	248	155	5	5	31
gi|354543410	hypothetical protein CPAR2_101670 [*Candida parapsilosis*]	13287	127	113	2	2	28
gi|354547945	hypothetical protein CPAR2_404840 [*Candida parapsilosis*]	21958	196	110	2	2	10
gi|354548524	hypothetical protein CPAR2_702740 [*Candida parapsilosis*]	17538	162	108	3	2	17

gi|354545766	hypothetical protein CPAR2_201370 [*Candida parapsilosis*]	33351	315	101	3	3	14
gi|354544553	hypothetical protein CPAR2_302650 [*Candida parapsilosis*]	230568	2047	93	3	3	2
gi|354546231	hypothetical protein CPAR2_206020 [*Candida parapsilosis*]	54125	485	90	3	3	9
gi|8927048	elongation factor 2 [*Candida parapsilosis*]	90305	813	81	2	2	4
gi|354545297	hypothetical protein CPAR2_805730 [*Candida parapsilosis*]	19579	177	71	1	1	7
anaerobic conditions (AN) 2							
i|354543937	hypothetical protein CPAR2_106940 [*Candida parapsilosis*]	69920	646	1164	26	19	40
gi|354548298	hypothetical protein CPAR2_700380 [*Candida parapsilosis*]	61254	568	838	20	15	33
gi|354546348	hypothetical protein CPAR2_207210 [*Candida parapsilosis*]	46995	439	802	15	12	41
gi|354545590	hypothetical protein CPAR2_808670 [*Candida parapsilosis*]	36264	336	729	19	13	53
gi|354543158	hypothetical protein CPAR2_602950 [*Candida parapsilosis*]	45057	417	650	18	12	41
gi|354547143	hypothetical protein CPAR2_501020 [*Candida parapsilosis*]	62596	566	495	11	9	27
gi|354548565	hypothetical protein CPAR2_703150 [*Candida parapsilosis*]	81506	710	341	11	10	20
gi|354547299	hypothetical protein CPAR2_502580 [*Candida parapsilosis*]	43513	23	321	7	7	19
gi|354546805	hypothetical protein CPAR2_211810 [*Candida parapsilosis*]	27649	248	316	10	7	47
gi|354547586	hypothetical protein CPAR2_401230 [*Candida parapsilosis*]	39816	359	311	9	9	28
gi|354545198	hypothetical protein CPAR2_804740 [*Candida parapsilosis*]	74602	681	234	9	8	19
gi|354545888	hypothetical protein CPAR2_202600 [*Candida parapsilosis*]	35921	324	219	3	3	12
gi|354546116	hypothetical protein CPAR2_204880 [*Candida parapsilosis*]	61499	549	216	6	6	15
gi|354543410	hypothetical protein CPAR2_101670 [*Candida parapsilosis*]	13287	127	186	4	4	51
gi|254558244	cytochrome P450 lanosterol 14a-demethylase [*Candida parapsilosis*]	59681	522	177	3	3	7
gi|354547945	hypothetical protein CPAR2_404840 [*Candida parapsilosis*]	21958	194	169	3	3	21
gi|354546327	hypothetical protein CPAR2_207000 [*Candida parapsilosis*]	47650	439	144	2	2	7
gi|354545980	hypothetical protein CPAR2_203520 [*Candida parapsilosis*]	55478	502	138	3	3	7
gi|354547336	hypothetical protein CPAR2_502950 [*Candida parapsilosis*]	98544	898	130	3	3	6
gi|354543177	hypothetical protein CPAR2_603140 [*Candida parapsilosis*]	44544	397	126	6	4	16
gi|354543792	hypothetical protein CPAR2_105500 [*Candida parapsilosis*]	33857	303	125	4	4	24
gi|354543405	hypothetical protein CPAR2_101620 [*Candida parapsilosis*]	57733	520	120	3	3	11

gi|354544553	hypothetical protein CPAR2_302650 [*Candida parapsilosis*]	230568	2047	110	5	5	4
gi|354548524	hypothetical protein CPAR2_702740 [*Candida parapsilosis*]	17538	162	103	4	3	20
gi|354548660	hypothetical protein CPAR2_704110 [*Candida parapsilosis*]	61779	550	101	3	3	5
gi|354545766	hypothetical protein CPAR2_201370 [*Candida parapsilosis*]	33351	315	97	2	2	9
gi|354545521	hypothetical protein CPAR2_807980 [*Candida parapsilosis*]	26955	248	96	3	3	25
gi|8927048	elongation factor 2 [*Candida parapsilosis*]	90305	813	88	2	2	4
gi|354544140	hypothetical protein CPAR2_109010 [*Candida parapsilosis*]	20285	181	82	2	2	16
gi|354543747	hypothetical protein CPAR2_105050 [*Candida parapsilosis*]	70207	651	78	2	2	4
gi|354548491	hypothetical protein CPAR2_702400 [*Candida parapsilosis*]	10054	87	70	2	2	25
gi|354547414	hypothetical protein CPAR2_503730 [*Candida parapsilosis*]	21149	186	68	2	2	13
anaerobic conditions (AN) 3							
gi|354543937	hypothetical protein CPAR2_106940 [*Candida parapsilosis*]	69920	646	1083	22	18	42
gi|354546348	hypothetical protein CPAR2_207210 [*Candida parapsilosis*]	46995	439	819	17	14	50
gi|354548298	hypothetical protein CPAR2_700380 [*Candida parapsilosis*]	61254	568	795	16	13	34
gi|354545590	hypothetical protein CPAR2_808670 [*Candida parapsilosis*]	36264	336	669	18	10	45
gi|354548565	hypothetical protein CPAR2_703150 [*Candida parapsilosis*]	81506	310	547	14	13	706
gi|354543158	hypothetical protein CPAR2_602950 [*Candida parapsilosis*]	45057	417	526	17	12	42
gi|354547143	hypothetical protein CPAR2_501020 [*Candida parapsilosis*]	62596	566	375	9	6	16
gi|354546805	hypothetical protein CPAR2_211810 [*Candida parapsilosis*]	27649	248	315	13	10	54
gi|354547586	hypothetical protein CPAR2_401230 [*Candida parapsilosis*]	39816	359	261	6	6	21
gi|354545198	hypothetical protein CPAR2_804740 [*Candida parapsilosis*]	74602	681	261	8	8	19
gi|354547336	hypothetical protein CPAR2_502950 [*Candida parapsilosis*]	98544	898	253	4	4	7
gi|354547299	hypothetical protein CPAR2_502580 [*Candida parapsilosis*]	43513	403	235	6	6	17
gi|354545980	hypothetical protein CPAR2_203520 [*Candida parapsilosis*]	55478	502	214	4	4	12
gi|354543410	hypothetical protein CPAR2_101670 [*Candida parapsilosis*]	13287	127	214	5	4	51
gi|354546116	hypothetical protein CPAR2_204880 [*Candida parapsilosis*]	61499	549	197	3	3	9
gi|354545521	hypothetical protein CPAR2_807980 [*Candida parapsilosis*]	26955	248	194	5	5	27
gi|354547945	hypothetical protein CPAR2_404840 [*Candida parapsilosis*]	21958	196	188	3	3	18
gi|354545888	hypothetical protein CPAR2_202600 [*Candida parapsilosis*]	35921	324	182	3	3	12

gi|354547001	hypothetical protein CPAR2_213780 [*Candida parapsilosis*]	74559	684	170	4	4	7
gi|354545766	hypothetical protein CPAR2_201370 [*Candida parapsilosis*]	33351	315	146	3	3	13
gi|9650661	actin [*Candida parapsilosis*]	36544	326	121	4	4	11
gi|354543792	hypothetical protein CPAR2_105500 [*Candida parapsilosis*]	33857	304	120	6	5	26
gi|354543405	hypothetical protein CPAR2_101620 [*Candida parapsilosis*]	57733	520	117	4	3	11
gi|354543177	hypothetical protein CPAR2_603140 [*Candida parapsilosis*]	44544	397	106	4	3	13
gi|354544140	hypothetical protein CPAR2_109010 [*Candida parapsilosis*]	20285	181	97	3	3	28
gi|3549613	ADP/ATP carrier protein [*Candida parapsilosis*]	32914	303	76	1	1	6
gi|354547561	hypothetical protein CPAR2_400980 [*Candida parapsilosis*]	20385	172	75	3	2	13
gi|8927048	elongation factor 2 [*Candida parapsilosis*]	90305	813	74	2	2	3
gi|354545297	hypothetical protein CPAR2_805730 [*Candida parapsilosis*]	19579	177	70	1	1	7

Score, the sum of the highest ions score for each distinct peptide sequence, excluding the scores of duplicate matches, the ions score for an MS/MS match is based on the calculated probability, P, that the observed match between the experimental data and the database sequence is a random event, the reported ion score is -10Log(P); **Matches**, the number of all peptides identified for a single protein in result report; **Sequences**, the number of different peptide sequences identified for a single protein in result report; **Sequence coverage**, the percentage coverage of the protein sequence by the identified peptides.
