# Supplementary material for: Moonlighting proteins are variably exposed at the cell surfaces of Candida glabrata, Candida parapsilosis and Candida tropicalis under certain growth conditions
Source: BMC Microbiol. 2019 Jul 3;19:149. doi: 10.1186/s12866-019-1524-5 (PMC6609379; doi:10.1186/s12866-019-1524-5)
Supplement: Supplementary file 4 — Table S4. Mass spectrometry identification of C. tropicalis proteins present at the cell surface under different growth conditions. (PDF 224 kb) [file 12866_2019_1524_MOESM4_ESM.pdf]

Supplementary table 4. Mass spectrometry identification of *C. tropicalis* proteins present at the cell surface under different growth conditions.

Cell surface shaving of fungal cells with trypsin and the additional digestion of the obtained proteins for 24 hours was performed. The resulting peptides were analyzed using the Dionex Ultimate 3000 UHPLC system coupled to an HCTUltra ETDII mass spectrometer. The obtained lists of peaks were searched against the NCBI protein database using an in-house Mascot server.

Accession number	Protein	Molecular mass [Da]	Number of amino acids	Score	Matches	Sequences	Sequence coverage [%]
defined synthetic medium (DS) 1							
gi|255727881	enolase 1 [*Candida tropicalis* MYA-3404]	46984	440	660	21	15	52
gi|255732890	glyceraldehyde-3-phosphate dehydrogenase [*Candida tropicalis* MYA-3404]	36209	335	570	18	16	53
gi|255729208	pyruvate decarboxylase [*Candida tropicalis* MYA-3404]	62500	537	558	13	9	27
gi|255722021	transaldolase [*Candida tropicalis* MYA-3404]	35641	333	360	6	6	28
gi|255721021	malate dehydrogenase, mitochondrial precursor [*Candida tropicalis* MYA-3404]	34822	332	310	7	7	32
gi|255733002	plasma membrane ATPase 1 [*Candida tropicalis* MYA-3404]	98358	895	304	6	6	10
gi|255730807	pyridoxine biosynthesis protein PDX1 [*Candida tropicalis* MYA-3404]	38483	343	276	4	4	14
gi|255727428	phosphoglycerate kinase [*Candida tropicalis* MYA-3404]	44686	416	260	6	6	21
gi|255731011	heat shock protein 70 2 [*Candida tropicalis* MYA-3404]	70144	644	251	5	5	10
gi|255728851	heat shock protein SSA1 [*Candida tropicalis* MYA-3404]	69963	648	239	6	6	15
gi|255725714	hypothetical protein CTRG_02093 [*Candida tropicalis* MYA-3404]	39082	370	235	5	4	19
gi|255722691	phosphoenolpyruvate carboxykinase [*Candida tropicalis* MYA-3404]	61920	554	212	4	4	9
gi|255722423	methionine-synthesizing 5-methyltetrahydropteroyltriglutamate-homocysteine methyltransferase [*Candida tropicalis* MYA-3404]	85686	767	208	5	5	8
gi|255720819	ATP synthase beta chain, mitochondrial precursor [*Candida tropicalis* MYA-3404]	44505	415	195	4	4	13
gi|255727773	heat shock protein SSB1 [*Candida tropicalis* MYA-3404]	66634	513	186	7	7	18
gi|255724622	40S ribosomal protein S8 [*Candida tropicalis* MYA-3404]	22506	202	180	4	4	29
gi|255729820	conserved hypothetical protein [*Candida tropicalis* MYA-3404]	35427	325	158	5	3	13
gi|255727899	transketolase 1 [*Candida tropicalis* MYA-3404]	73908	677	158	4	4	9

gi|255721795	heat shock protein 60, mitochondrial precursor [*Candida tropicalis* MYA-3404]	60491	569	155	3	3	7
gi|255732521	alcohol dehydrogenase 2 [*Candida tropicalis* MYA-3404]	37181	348	139	4	4	11
gi|255732093	ADP,ATP carrier protein [*Candida tropicalis* MYA-3404]	33532	308	133	3	3	11
gi|255723882	phosphoglycerate mutase 1 [*Candida tropicalis* MYA-3404]	27491	248	123	3	3	20
gi|255729756	S-adenosylmethionine synthetase [*Candida tropicalis* MYA-3404]	42576	385	114	2	2	6
gi|255730713	hypothetical protein CTRG_04579 [*Candida tropicalis* MYA-3404]	125610	1156	111	4	4	4
gi|255724456	60S ribosomal protein L31 [*Candida tropicalis* MYA-3404]	13005	112	111	2	2	22
gi|255732774	triosephosphate isomerase [*Candida tropicalis* MYA-3404]	26769	248	109	2	2	14
gi|255726006	peroxiredoxin TSA1 [*Candida tropicalis* MYA-3404]	21839	195	99	3	3	18
gi|255722063	citrate synthase, mitochondrial precursor [*Candida tropicalis* MYA-3404]	52018	467	95	4	3	8
gi|255723403	alcohol dehydrogenase 1 [*Candida tropicalis* MYA-3404]	43927	408	90	2	2	5
gi|255725240	predicted protein [*Candida tropicalis* MYA-3404]	66682	631	89	2	2	3
gi|255730149	protein EPD1 precursor [*Candida tropicalis* MYA-3404]	59740	552	86	2	2	5
gi|255726604	hypothetical protein CTRG_02525 [*Candida tropicalis* MYA-3404]	35772	329	75	2	1	6
gi|255722832	inositol-3-phosphate synthase [*Candida tropicalis* MYA-3404]	57018	517	71	4	4	12
gi|255727689	heat shock protein 104 [*Candida tropicalis* MYA-3404]	100361	901	69	1	1	1
defined synthetic medium (DS) 2							
gi|255727881	enolase 1 [*Candida tropicalis* MYA-3404]	46984	440	811	19	15	42
gi|255729208	pyruvate decarboxylase [*Candida tropicalis* MYA-3404]	62500	567	567	14	12	35
gi|255732890	glyceraldehyde-3-phosphate dehydrogenase [*Candida tropicalis* MYA-3404]	36209	336	474	15	13	45
gi|255722021	transaldolase [*Candida tropicalis* MYA-3404]	35641	323	420	8	8	34
gi|255721021	malate dehydrogenase, mitochondrial precursor [*Candida tropicalis* MYA-3404]	34822	332	391	8	8	36
gi|255731011	heat shock protein 70 2 [*Candida tropicalis* MYA-3404]	70144	644	305	6	5	10
gi|255727428	phosphoglycerate kinase [*Candida tropicalis* MYA-3404]	44686	416	289	8	7	21
gi|255721795	heat shock protein 60, mitochondrial precursor [*Candida tropicalis* MYA-3404]	60491	569	285	4	4	10
gi|255720819	ATP synthase beta chain, mitochondrial precursor [*Candida tropicalis* MYA-3404]	44505	415	274	6	5	16
gi|255728851	heat shock protein SSA1 [*Candida tropicalis* MYA-3404]	69963	648	268	7	7	16
gi|255725714	hypothetical protein CTRG_02093 [*Candida tropicalis* MYA-3404]	39082	370	260	6	4	19

gi|255730807	pyridoxine biosynthesis protein PDX1 [*Candida tropicalis* MYA-3404]	38483	343	209	3	3	15
gi|255729820	conserved hypothetical protein [*Candida tropicalis* MYA-3404]	35427	325	206	5	4	16
gi|255727773	heat shock protein SSB1 [*Candida tropicalis* MYA-3404]	66634	613	198	5	5	9
gi|255733002	plasma membrane ATPase 1 [*Candida tropicalis* MYA-3404]	98358	895	188	5	5	9
gi|255730951	ATP synthase alpha chain, mitochondrial precursor [*Candida tropicalis* MYA-3404]	48616	447	183	6	6	13
gi|255725194	elongation factor 1-alpha [*Candida tropicalis* MYA-3404]	50336	458	180	4	4	10
gi|255723882	phosphoglycerate mutase 1 [*Candida tropicalis* MYA-3404]	27491	248	149	4	3	19
gi|255725884	40S ribosomal protein S7-A [*Candida tropicalis* MYA-3404]	21189	186	143	3	3	19
gi|255732774	triosephosphate isomerase [*Candida tropicalis* MYA-3404]	26769	248	141	4	3	20
gi|255722691	phosphoenolpyruvate carboxykinase [*Candida tropicalis* MYA-3404]	61920	554	128	3	3	6
gi|255722832	inositol-3-phosphate synthase [*Candida tropicalis* MYA-3404]	57018	517	127	6	6	18
gi|255730713	hypothetical protein CTRG_04579 [*Candida tropicalis* MYA-3404]	125610	1156	126	5	5	5
gi|255727899	transketolase 1 [*Candida tropicalis* MYA-3404]	73908	677	115	2	2	4
gi|255726132	60S ribosomal protein L19 [*Candida tropicalis* MYA-3404]	20009	172	111	2	2	10
gi|255725240	predicted protein [*Candida tropicalis* MYA-3404]	66682	631	106	2	2	3
gi|255732521	alcohol dehydrogenase 2 [*Candida tropicalis* MYA-3404]	37181	348	102	3	3	9
gi|255723010	pyrimidine precursor biosynthesis enzyme THI12 [*Candida tropicalis* MYA-3404]	38967	341	86	3	3	18
gi|255726338	60S ribosomal protein L12 [*Candida tropicalis* MYA-3404]	17800	165	84	1	1	9
gi|255720963	60S ribosomal protein L20 [*Candida tropicalis* MYA-3404]	20352	172	79	2	2	11
gi|255725262	60S ribosomal protein L3 [*Candida tropicalis* MYA-3404]	44049	389	79	1	1	3
gi|255720991	fructose-bisphosphate aldolase [*Candida tropicalis* MYA-3404]	33867	311	77	2	2	8
gi|255724622	40S ribosomal protein S8 [*Candida tropicalis* MYA-3404]	22506	202	69	2	2	13
gi|255724456	60S ribosomal protein L31 [*Candida tropicalis* MYA-3404]	13005	112	68	2	2	22
gi|7549229	heat shock protein 90 [*Candida tropicalis*]	78939	690	68	2	2	3
gi|255720621	40S ribosomal protein S6 [*Candida tropicalis* MYA-3404]	27170	246	67	1	1	5
gi|255730231	60S ribosomal protein L13 [*Candida tropicalis* MYA-3404]	23082	202	67	2	2	9
defined synthetic medium (DS) 3							
gi|255727881	enolase 1 [*Candida tropicalis* MYA-3404]	46984	440	588	18	15	54
gi|255732890	glyceraldehyde-3-phosphate dehydrogenase [*Candida tropicalis* MYA-3404]	36209	336	583	16	12	51

gi|255721021	malate dehydrogenase, mitochondrial precursor [*Candida tropicalis* MYA-3404]	34822	332	410	10	8	35
gi|255720819	ATP synthase beta chain, mitochondrial precursor [*Candida tropicalis* MYA-3404]	44505	415	405	9	8	31
gi|255722021	transaldolase [*Candida tropicalis* MYA-3404]	35641	323	363	8	7	32
gi|255728851	heat shock protein SSA1 [*Candida tropicalis* MYA-3404]	69963	648	350	9	9	21
gi|255729208	pyruvate decarboxylase [*Candida tropicalis* MYA-3404]	62500	467	313	7	6	22
gi|255721795	heat shock protein 60, mitochondrial precursor [*Candida tropicalis* MYA-3404]	60491	569	297	5	5	13
gi|255731011	heat shock protein 70 2 [*Candida tropicalis* MYA-3404]	70144	644	284	8	8	21
gi|255725194	elongation factor 1-alpha [*Candida tropicalis* MYA-3404]	50336	458	255	9	9	24
gi|255726604	hypothetical protein CTRG_02525 [*Candida tropicalis* MYA-3404]	35772	329	253	5	4	19
gi|255726556	fatty acid synthase alpha subunit [*Candida tropicalis* MYA-3404]5.38	207017	1883	251	7	7	
gi|255726006	peroxiredoxin TSA1 [*Candida tropicalis* MYA-3404]	21839	196	215	6	6	32
gi|255727773	heat shock protein SSB1 [*Candida tropicalis* MYA-3404]	66634	613	214	9	9	23
gi|255726910	elongation factor 3 [*Candida tropicalis* MYA-3404]	117062	1050	211	4	4	5
gi|255725884	40S ribosomal protein S7-A [*Candida tropicalis* MYA-3404]	21189	186	207	4	4	26
gi|255730807	pyridoxine biosynthesis protein PDX1 [*Candida tropicalis* MYA-3404]	38483	343	201	3	3	15
gi|255727362	conserved hypothetical protein [*Candida tropicalis* MYA-3404]	18822	174	191	5	4	33
gi|255723882	phosphoglycerate mutase 1 [*Candida tropicalis* MYA-3404]	27491	248	175	4	4	31
gi|255733002	plasma membrane ATPase 1 [*Candida tropicalis* MYA-3404]	98358	895	174	5	5	7
gi|255722063	citrate synthase, mitochondrial precursor [*Candida tropicalis* MYA-3404]	52018	467	173	6	5	21
gi|255722423	methionine-synthesizing 5-methyltetrahydropteroyltriglutamate-homocysteine methyltransferase [*Candida tropicalis* MYA-3404]	85686	767	165	6	6	12
gi|255725714	hypothetical protein CTRG_02093 [*Candida tropicalis* MYA-3404]	39082	370	164	5	4	19
gi|255728875	6-phosphogluconate dehydrogenase [*Candida tropicalis* MYA-3404]	56787	515	148	3	3	8
gi|255730951	ATP synthase alpha chain, mitochondrial precursor [*Candida tropicalis* MYA-3404]	48616	447	147	5	5	14
gi|8927044	elongation factor 2 [*Candida tropicalis*]	90177	813	132	4	3	6
gi|255720991	fructose-bisphosphate aldolase [*Candida tropicalis* MYA-3404]	33867	311	124	2	2	12
gi|255728927	60S ribosomal protein L4-B [*Candida tropicalis* MYA-3404]	39189	363	123	5	5	23
gi|255726586	60S ribosomal protein L8-B [*Candida tropicalis* MYA-3404]	28289	261	122	4	3	17
gi|255722691	phosphoenolpyruvate carboxykinase [*Candida tropicalis* MYA-3404]	61920	554	120	3	3	8

gi|255730231	60S ribosomal protein L13 [*Candida tropicalis* MYA-3404]	23082	202	117	2	2	10
gi|255724698	acetyl-CoA acetyltransferase IB [*Candida tropicalis* MYA-3404]	42032	403	113	3	3	13
gi|255732093	ADP,ATP carrier protein [*Candida tropicalis* MYA-3404]	33532	308	112	3	3	13
gi|255727394	60S ribosomal protein L10a [*Candida tropicalis* MYA-3404]	24468	217	112	2	2	12
gi|9650655	actin [*Candida tropicalis*]	36614	326	110	5	5	27
gi|255720963	60S ribosomal protein L20 [*Candida tropicalis* MYA-3404]	20352	172	95	2	2	13
gi|255724654	ATP synthase subunit 4, mitochondrial precursor [*Candida tropicalis* MYA-3404]	25946	233	91	2	2	8
gi|255731328	60S acidic ribosomal protein P0 [*Candida tropicalis* MYA-3404]	33485	313	84	3	3	13
gi|255722832	inositol-3-phosphate synthase [*Candida tropicalis* MYA-3404]	57018	517	84	4	4	12
gi|255727428	phosphoglycerate kinase [*Candida tropicalis* MYA-3404]	44686	416	83	3	3	11
gi|255722577	argininosuccinate synthase [*Candida tropicalis* MYA-3404]	46197	416	80	3	2	9
gi|255732039	fatty acid synthase beta subunit dehydratase [*Candida tropicalis* MYA-3404]	229051	2039	80	2	2	1
gi|255732521	alcohol dehydrogenase 2 [*Candida tropicalis* MYA-3404]	37181	348	72	2	2	11
gi|255720621	40S ribosomal protein S6 [*Candida tropicalis* MYA-3404]	27170	236	68	1	1	5
gi|255731334	40S ribosomal protein S18 [*Candida tropicalis* MYA-3404]	15667	133	68	1	1	8
artificial saliva (AS) 1							
gi|255728723	opaque-phase-specific protein OP4 precursor [*Candida tropicalis* MYA-3404]	43376	421	127	2	2	6
gi|255727881	enolase 1 [*Candida tropicalis* MYA-3404]	46984	440	110	3	3	10
gi|255732890	glyceraldehyde-3-phosphate dehydrogenase [*Candida tropicalis* MYA-3404]	36209	336	75	4	4	14
gi|255722691	phosphoenolpyruvate carboxykinase [*Candida tropicalis* MYA-3404]	61920	554	72	3	3	8
artificial saliva (AS) 2							
gi|255728723	opaque-phase-specific protein OP4 precursor [*Candida tropicalis* MYA-3404]	43376	421	186	3	3	9
gi|255721523	predicted protein [*Candida tropicalis* MYA-3404]	98965	929	101	3	3	4
gi|255727881	enolase 1 [*Candida tropicalis* MYA-3404]	46984	440	87	2	2	6
artificial saliva (AS) 3							
gi|255725714	hypothetical protein CTRG_02093 [*Candida tropicalis* MYA-3404]	39082	370	232	5	4	19
gi|255721523	predicted protein [*Candida tropicalis* MYA-3404]	98965	929	205	5	3	4
gi|255731223	conserved hypothetical protein [*Candida tropicalis* MYA-3404]	38035	355	89	3	2	11
gi|255722347	hypothetical protein CTRG_00890 [*Candida tropicalis* MYA-3404]	53021	465	76	2	2	5

gi|255732521	alcohol dehydrogenase 2 [*Candida tropicalis* MYA-3404]	37181	348	76	2	2	5
vagina-simulative medium (VS) 1							
gi|255722347	hypothetical protein CTRG_00890 acid phosphatase Pho113 [*Candida tropicalis* MYA-3404]	53021	455	218	6	3	8
gi|255732890	glyceraldehyde-3-phosphate dehydrogenase [*Candida tropicalis* MYA-3404]	36209	336	209	12	6	28
gi|255725714	hypothetical protein CTRG_02093 MP65 [*Candida tropicalis* MYA-3404]	39082	370	122	4	2	9
vagina-simulative medium (VS) 2							
gi|255722347	hypothetical protein CTRG_00890 [*Candida tropicalis* MYA-3404]	53021	465	187	3	3	8
gi|255732890	glyceraldehyde-3-phosphate dehydrogenase [*Candida tropicalis* MYA-3404]	36209	336	183	5	5	25
gi|255725714	hypothetical protein CTRG_02093 [*Candida tropicalis* MYA-3404]	39082	370	117	2	2	9
vagina-simulative medium (VS) 3							
gi|255722347	hypothetical protein CTRG_00890 [*Candida tropicalis* MYA-3404]	53021	465	262	4	4	11
gi|255731223	conserved hypothetical protein [*Candida tropicalis* MYA-3404]	38035	356	244	6	4	20
gi|255725714	hypothetical protein CTRG_02093 [*Candida tropicalis* MYA-3404]	39082	370	180	2	2	9
artificial urine (AU) 1							
gi|255722063	citrate synthase, mitochondrial precursor [*Candida tropicalis* MYA-3404]	52018	467	208	5	5	13
gi|255727881	enolase 1 [*Candida tropicalis* MYA-3404]	46984	440	153	3	3	10
artificial urine (AU) 2							
gi|255727881	enolase 1 [*Candida tropicalis* MYA-3404]	46984	440	117	6	5	17
gi|255727428	phosphoglycerate kinase [*Candida tropicalis* MYA-3404]	44686	416	113	5	4	9
gi|255731950	alcohol dehydrogenase 2 [*Candida tropicalis* MYA-3404]	37165	348	93	3	2	6
gi|255723403	alcohol dehydrogenase 1 [*Candida tropicalis* MYA-3404]	43927	408	91	4	3	8
gi|255725194	elongation factor 1-alpha [*Candida tropicalis* MYA-3404]	50336	458	78	4	4	8
anaerobic conditions (AN) 1							
gi|255721523	predicted protein [*Candida tropicalis* MYA-3404]	98965	929	247	4	3	4
gi|255725714	hypothetical protein CTRG_02093 [*Candida tropicalis* MYA-3404]	39082	370	197	5	3	16
gi|255727881	enolase 1 [*Candida tropicalis* MYA-3404]	46984	440	127	9	7	33
gi|255722814	thioredoxin II [*Candida tropicalis* MYA-3404]	11597	105	102	2	2	23
gi|255725240	predicted protein [*Candida tropicalis* MYA-3404]	66682	631	87	2	2	3

gi|255730149	protein EPD1 precursor [*Candida tropicalis* MYA-3404]	59740	552	73	2	2	4
gi|255723403	alcohol dehydrogenase 1 [*Candida tropicalis* MYA-3404]	43927	408	71	2	2	5
anaerobic conditions (AN) 2							
gi|255725714	hypothetical protein CTRG_02093 MP65 [*Candida tropicalis* MYA-3404]	39082	370	300	6	5	19
gi|255727881	enolase 1 [*Candida tropicalis* MYA-3404]	46984	440	219	9	8	35
gi|255721523	predicted protein [*Candida tropicalis* MYA-3404]	98965	929	204	4	3	4
gi|255722814	thioredoxin II [*Candida tropicalis* MYA-3404]	11597	105	156	5	4	42
gi|255731742	FK506-binding protein [*Candida tropicalis* MYA-3404]	12991	121	146	2	2	21
gi|255729820	conserved hypothetical protein [*Candida tropicalis* MYA-3404]	35427	326	135	4	3	13
gi|255732890	glyceraldehyde-3-phosphate dehydrogenase [*Candida tropicalis* MYA-3404]	36209	336	117	4	3	16
gi|255725238	predicted protein [*Candida tropicalis* MYA-3404]	110185	1065	112	5	4	4
gi|255720853	hypothetical protein CTRG_00142 [*Candida tropicalis* MYA-3404]	19174	173	110	2	2	20
gi|255730149	protein EPD1 precursor [*Candida tropicalis* MYA-3404]	59740	552	96	2	2	4
gi|255725240	predicted protein [*Candida tropicalis* MYA-3404]	66682	631	90	2	1	2
gi|255723990	hypothetical protein CTRG_01230 [*Candida tropicalis* MYA-3404]	75381	679	74	1	1	1
gi|255729208	pyruvate decarboxylase [*Candida tropicalis* MYA-3404]	62500	567	69	3	3	10
anaerobic conditions (AN) 3							
gi|255725714	hypothetical protein CTRG_02093 [*Candida tropicalis* MYA-3404]	39082	370	264	7	5	19
gi|255721523	predicted protein [*Candida tropicalis* MYA-3404]	98965	929	217	4	3	4
gi|255722814	thioredoxin II [*Candida tropicalis* MYA-3404]	11597	105	177	4	4	42
gi|255732890	glyceraldehyde-3-phosphate dehydrogenase [*Candida tropicalis* MYA-3404]	36209	336	125	5	5	29
gi|255731742	FK506-binding protein [*Candida tropicalis* MYA-3404]	12991	121	123	3	3	34
gi|255729820	conserved hypothetical protein [*Candida tropicalis* MYA-3404]]	35427	325	109	3	2	10
gi|255725240	predicted protein [*Candida tropicalis* MYA-3404]	66682	631	105	2	2	3
gi|255723990	hypothetical protein CTRG_01230 [*Candida tropicalis* MYA-3404]	75381	679	103	2	2	3
gi|255727881	enolase 1 [*Candida tropicalis* MYA-3404]	46984	440	76	3	2	8
gi|255729208	pyruvate decarboxylase [*Candida tropicalis* MYA-3404]	62500	567	68	3	2	7

Score, the sum of the highest ions score for each distinct peptide sequence, excluding the scores of duplicate matches, the ions score for an MS/MS match is based on the calculated probability, P, that the observed match between the experimental data and the database sequence is a random event, the reported ion score is -10Log(P); **Matches**, the number of all peptides identified for a single protein in result report; **Sequences**, the number of different peptide sequences identified for a single protein in result report; **Sequence coverage**, the percentage coverage of the protein sequence by the identified peptides.
