# Supplementary figures and images for: Moonlighting proteins are variably exposed at the cell surfaces of Candida glabrata, Candida parapsilosis and Candida tropicalis under certain growth conditions
Source: BMC Microbiol. 2019 Jul 3;19:149. doi: 10.1186/s12866-019-1524-5 (PMC6609379; doi:10.1186/s12866-019-1524-5)

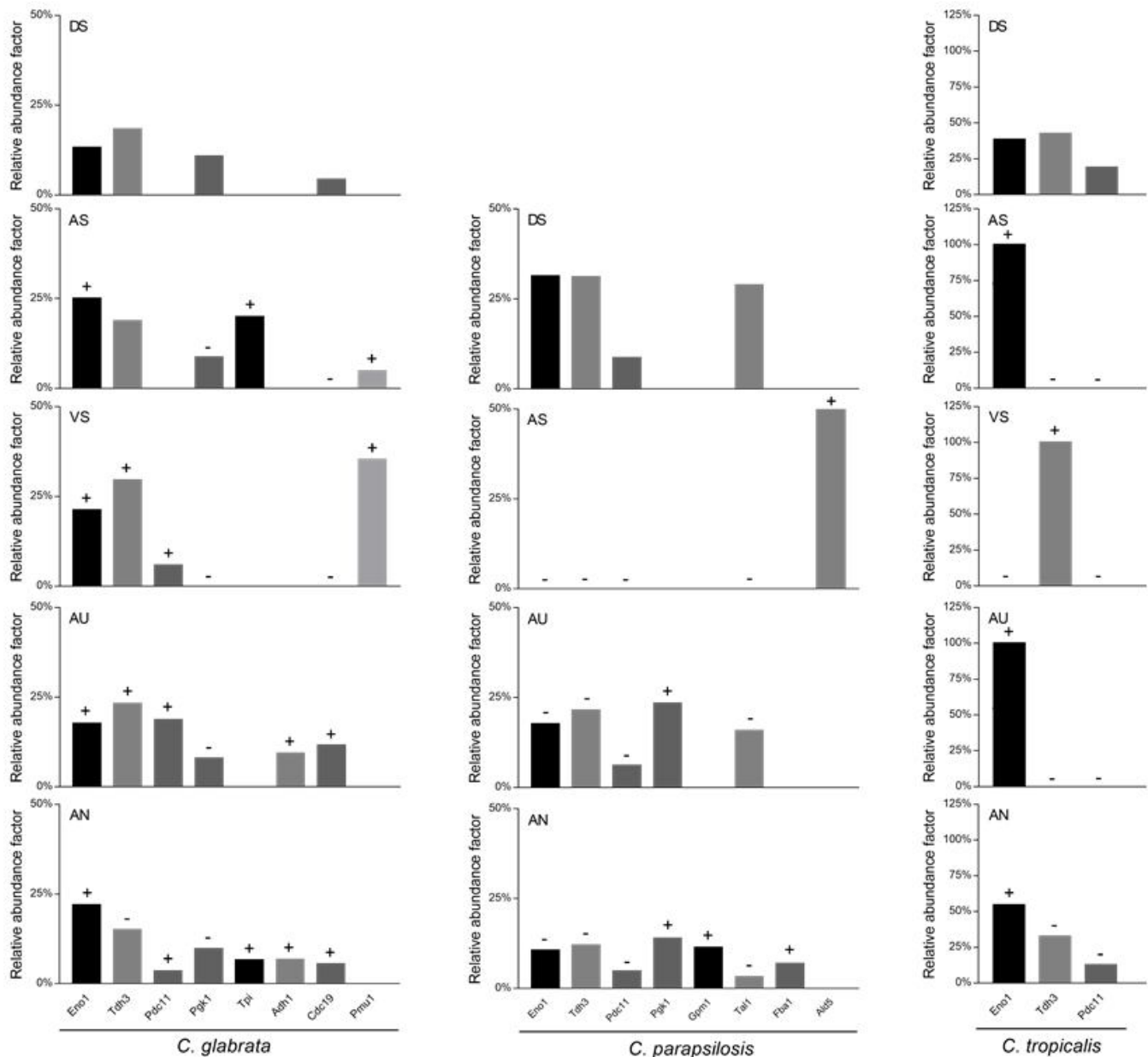

Supplement: Supplementary file 5 — Figure S1. Relative differences in the level of expression of selected surface-exposed moonlighting proteins depending on the type of medium used. (PDF 171 kb) [file 12866_2019_1524_MOESM5_ESM.pdf]
